# Screening and characterization of a novel cupin-like polypeptide from Oyster (*Ostrea gigas*) and its anti-inflammatory effect via TAK1 mediated NF-κB pathway

**DOI:** 10.1016/j.fochx.2026.103884

**Published:** 2026-04-21

**Authors:** Chunlei Li, Yanxiao Xiang, Tao Jiang, Jiyuan Zhang, Xuekui Xia, Anchang Liu

**Affiliations:** a Department of Pharmacy, Qilu Hospital, Cheeloo College of Medicine, Shandong University, Jinan 250012, China; b Shandong Provincial Key Laboratory for Bio-Manufacturing, Biology Institute, Qilu University of Technology (Shandong Academy of Sciences), Jinan 250103, China; c Department of Clinical Pharmacy, School of Pharmaceutical Sciences, Shandong University, Jinan, China

**Keywords:** *Ostrea gigas*, Polypeptide, Anti-inflammatory activity, TAK1, Cupin domain

## Abstract

Inflammation is a natural host defense response to injury or infection, and seafood-derived polypeptides exhibit prominent anti-inflammatory potential with promising therapeutic applications. Here, we report the screening, characterization and anti-inflammatory evaluation of a novel cupin-like polypeptide OGP1-1 from *Ostrea gigas*. OGP1-1 exerted significant anti-inflammatory effects in RAW264.7 macrophages in vitro and in a transgenic zebrafish model in vivo. Chemical proteomics identified transforming growth factor-β-activated kinase 1 (TAK1) as the direct molecular target, and molecular docking and dynamics simulations confirmed stable binding within TAK1's activation loop. Mechanistic studies demonstrated that OGP1-1 exerts its anti-inflammatory effects by suppressing the TAK1-mediated NF-κB signaling pathway. Collectively, these findings position OGP1-1 as a novel cupin-like polypeptide with potent anti-inflammatory activity and high potential for development as a functional food ingredient in inflammation management.

## Highlights

• OGP1-1 was discovered from oyster, Ostrea gigas, as a cupin-like polypeptide.
• OGP1-1 exhibited anti-inflammatory activities both in vitro and in vivo.
• Chemical proteomics and molecular docking revealed OGP1-1 binds to TAK1 protein.
• OGP1-1 suppressed TAK1-mediated activation of the MAPK and NF-κB pathways.

## Graphical abstract

**Figure:**
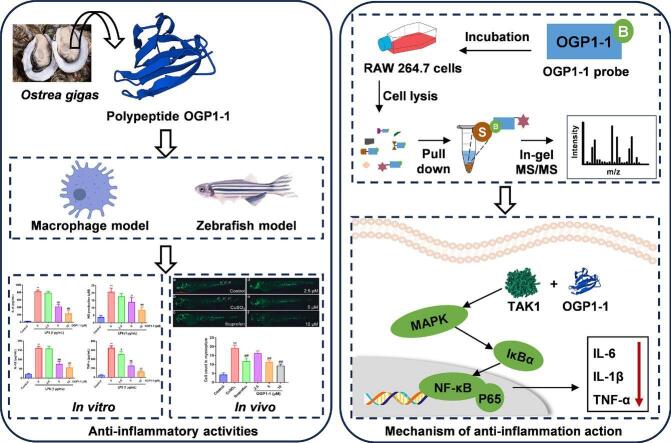

## 1 Introduction

Inflammation is an important progressive process, primarily comprising defensive responses, that takes place following bodily injury by inflammatory agents. Chronic inflammation disrupts the host's immunological tolerance, leading to the activation of pertinent immune cells that generate inflammatory mediators, including cytokines, prostaglandins, arachidonic acid, and histamine (Hamidzadeh et al., 2017; Rogovskii, 2020). Inflammatory mediators activate intracellular and extracellular inflammatory signaling pathways, leading immune cells to generate excessive quantities of IL-1β, COX-2, TNF-α, and other pro-inflammatory cytokines (Hamidzadeh et al., 2017; Turner et al., 2014). Transforming growth factor-β (TGF-β)-activated kinase 1 (TAK1), belonging to the mitogen-activated protein kinase kinase kinase (MAP3K) family, serves as a pivotal regulator within inflammatory signaling cascades (Xu & Lei, 2020). Its enzymatic function is potently stimulated by multiple pro-inflammatory mediators, including IL-1β, TNF-α, TGF-β, and COX-2 (Xu et al., 2020). Furthermore, TAK1 critically modulates the JNK and p38 MAPK pathways through phosphorylation-mediated activation of downstream kinases MKK4 and MKK3/6 (Zhao et al., 2023). Achieving full kinase activity necessitates interaction with its binding partner TAB1, which facilitates autophosphorylation at specific residues (Thr184, Thr187, and Ser192) within TAK1's activation loop via direct domain binding (Huang et al., 2025). Emerging evidence positions TAK1 as a central mediator in the pathogenesis of various inflammatory conditions, including autoimmune and neuroinflammatory disorders. Its ability to coordinate key processes, such as inflammatory signaling, tissue remodeling, apoptosis, proliferation, and cellular stress adaptation, highlights its therapeutic potential. Naturally derived small-molecule inhibitors, including 5Z-7-oxozeaenol and takinib, have shown promising results in preclinical models by selectively suppress TAK1 activation and mitigating inflammation-driven pathologies (Benedik et al., 2025; Totzke et al., 2017). In addition to these targeted synthetic inhibitors, glucocorticoids and nonsteroidal anti-inflammatory drugs (NSAIDs), which are widely used clinically, have also been found to inactivate TAK1 (Bhattacharyya et al., 2010; Shin et al., 2011). Nevertheless, adverse effects of glucocorticoids and NSAIDs, including Cushing's syndrome, coagulation, and allergic reactions—may lead to impaired immunity and metabolic dysregulation in individuals (Panchal & Prince Sabina, 2023). Consequently, TAK1 has garnered significant interest as a promising target for the treatment of inflammatory diseases, with significant efforts focused on developing novel and safe inhibitors derived from natural molecules. Notably, despite the therapeutic potential of natural polypeptides—known for their high specificity and relatively low toxicity—the discovery and characterization of polypeptide inhibitors targeting TAK1 remain significantly underreported, representing a distinct gap in the field.

In parallel, the search for safe and effective anti-inflammatory agents has turned increasingly towards marine organisms, which represent a vast and underexplored reservoir of structurally unique molecules due to their extreme habitats. (Cheung et al., 2016; Shin et al., 2011). Compared with terrestrial organisms, marine organisms cultivated in special ecological environments not only have distinct morphological features but are also different in metabolic and defense systems. Therefore, substantial quantities of bioactive molecules, including macrolides, terpenes, polypeptides, and polysaccharides with distinctive structures and functions, are produced and accumulated in marine organisms. Polypeptides extracted from marine organisms, especially seafood, have attracted significant attention due to their distinct anti-inflammatory properties (Ahmad et al., 2018). Three polypeptides, exhibiting significant anti-inflammatory properties, were isolated from sea cucumber (Z. Zhang et al., 2026). Furthermore, two peptides, PAY and SPHF1, were purified from salmon by-product hydrolysate, demonstrating notable anti-inflammatory properties (Ahn et al., 2012; Ahn et al., 2015). These peptides can obstruct the NO/iNOS and PGE2/COX-2 pathways, hence restricting the synthesis of pro-inflammatory cytokines, including TNF-α and IL-1β in RAW264.7 macrophages (Ahn et al., 2012; Ahn et al., 2015). Recently, several anti-inflammatory peptides were identified in the seahorses *Hippocampus*, a famous seafood in east Asia (Z. Zhang et al., 2024). Furthermore, a category of neurotoxic peptides called conotoxins was identified from marine mollusks, specifically sea snails. Conotoxins can disrupt pain signals and reduce inflammation by inhibiting or modifying the activation of nicotinic acetylcholine receptors and calcium channels (Holmes, 2014; Kutzsche et al., 2024). Ziconotide, a synthetic polypeptide derived from the natural ω-conotoxin MVIIA purified from the venom of *Conus magus*, has received FDA approval as a medicinal agent for alleviating severe chronic inflammatory pain (Kutzsche et al., 2024). These findings have stimulated growing interest in recovery and application of seafood-derived polypeptides, highlighting the potential to develop sustainable and novel sources of anti-inflammatory molecules for promoting health and wellness.

Oyster (*Ostrea gigas*) is an economically significant shellfish, widely distributed in the coastal countries of the Asia - Pacific region. Owing to its delicious flavour and good economic value, it has been cultivated for many years (Miao et al., 2018). Oyster has extensive applications in Chinese medicine and is often used in conjunction with other herbs in formulations to address symptoms, including palpitations, insomnia, dizziness, tinnitus, scrofula, subcutaneous nodules, and abdominal masses (Guan & Wang, 2009). The pharmacological effects of oysters include anti-fatigue, antitumor, anti-gastric ulcer, sedative, anti-inflammatory and anti-viral properties (Guan et al., 2009). The oyster can regulate diverse physiological processes and is included in the list of foods having medicinal and edible homology published by the Ministry of Health of China (C. P. Zhang et al., 2014). Recent investigations have demonstrated that oyster peptides possess anti-inflammatory properties. Oyster low-molecular-weight peptide extracts have shown the capacity to reduce inflammation induced by lipopolysaccharide (LPS) in acute colitis (Wu et al., 2024). Furthermore, several oligopeptides derived from oyster meat hydrolysates, including tyrosine-alanine (YA), threonine-tryptophan-proline (TWP), threonine-alanine-methionine-tyrosine (TAMY), and phenylalanine-proline-glycine-alanine (FPGA), demonstrated anti-inflammatory properties by effectively regulating the release of inflammatory mediators (Shen et al., 2023; Siregar et al., 2021). Hence, oysters are prioritized for research due to substantial economic value, medicinal utility in traditional Chinese medicine, and validated anti-inflammatory activities from peptide extracts documented in prior studies (Guan et al., 2009; Shen et al., 2023; Siregar et al., 2021; Wu et al., 2024). However, current research on oyster-derived anti-inflammatory peptides has predominantly focused on short oligopeptides. Consequently, the potential of larger, structurally defined polypeptides remains largely unexplored, representing a significant knowledge gap.

In recent years, the zebrafish (*Danio rerio*) model has emerged as a valuable in vivo platform for evaluating the anti-inflammatory activity of food-derived bioactive compounds. This model offers several distinct advantages over traditional mammalian systems, including high fecundity, optical transparency during early developmental stages, and a high degree of genetic and physiological conservation with humans (Gut et al., 2017). Notably, transgenic zebrafish line, Tg(Lyz: EGFP), with fluorescently labeled immune cells enable real-time visualization of macrophage and neutrophil migration in response to inflammatory stimuli, providing a direct and intuitive readout of anti-inflammatory efficacy (Olivari et al., 2008). The rapid development, low cost, and compliance with 3R (Replacement, Reduction, Refinement) principles further support the use of zebrafish as an initial in vivo screening model for functional food ingredients.

The elucidation of molecular mechanisms underlying food-derived bioactive molecules has increasingly relied on computational approaches, which have now become indispensable in this field. Among these, molecular docking and molecular dynamics simulations allow for the prediction and characterization of intermolecular interactions at atomic resolution. Molecular docking facilitates rapid screening of potential binding sites and conformations, while molecular dynamics simulations provide dynamic insights into the stability and energetics of ligand–receptor complexes under near-physiological conditions (Aguiar & Camps, 2025; Hou et al., 2025). These in silico methods not only accelerate target identification but also offer mechanistic insights that guide experimental validation, making them particularly valuable for studying polypeptide–protein interactions where structural complexity is high.

This study details the purification and physicochemical examination of a novel polypeptide, OGP1-1, derived from *Ostrea gigas*, as part of our ongoing research into bioactive polypeptides from Ostrea species. The in vitro anti-inflammatory efficacy and molecular mechanism of action of OGP-1 were investigated. The in vivo anti-inflammatory activity was evaluated using a zebrafish inflammation model. Moreover, through a combination of chemical proteomics and computational simulations, the direct target and the underlying mechanism were further elucidated.

## 2 Materials and methods

### 2.1 Materials

The oysters (*Ostrea gigas*) were obtained from Qingdao, Shandong province, China. All chemicals used were of analytical grade. The visceral mass was excised from *Ostrea gigas*, weighed, and preserved at −20 °C until used. The zebrafish used in this experiment are transgenic zebrafish tagged with green fluorescent, namely macrophage Tg (Lyz: EGFP). The fish are cultivated independently according to distinct strains and segregated by sex in a special zebrafish breeding house, fed with brine shrimp and pellets regularly. The ambient temperature is regulated at 28 ± 0.5 °C by the air conditioning system.

Commercial kits for RNA extraction, BCA assays, ROS assays, nitric oxide (NO) assays and ELISA were acquired from Beyotime (Shanghai, China). Trizol and phenylmethanesulfonyl fluoride (PMSF) were provided by Aladdin (Shanghai, China). Proteintech (Chicago, Illinois, USA) supplied antibodies, including anti-P38, anti-p-P38, anti-JNK, anti-p-JNK, anti-ERK, anti-p-ERK, anti-TLR-4, anti-NF-κB p65, anti-p-NF-κB p65, anti-IκB, anti-p-IκB, and anti-β-actin. Anti-COX-2 and anti-NOS2 antibodies were obtained from Cell Signaling Technology (Danvers, MA, USA). Acetonitrile, trifluoroacetic acid (TFA), Tris, Sodium dodecyl sulfate (SDS), bovine serum albumin (BSA), glucose, Coomassie brilliant blue R-250, DL-Dithiothreitol (DTT), Iodoacetamide (IAM), dimethyl sulfoxide (DMSO), tricaine, and phenylthiourea were procured from Sigma-Aldrich (St. Louis, MA, USA). Ibuprofen was purchased from the China Institute for Food and Drug Control, while the FastPure cell/tissue total RNA extraction kit, AceQ qPCR SYBR green master mix, and HiScript II Q RT SuperMix for qPCR with gDNA wiper were sourced from Vazyme Corporation (Nanjing, China). The One-Step PrimeScript RT-PCR Kit was obtained from Takara Corporation (Shiga, Japan). Trypsin was MS grade and acquired from Thermo Fisher (Waltham, USA.). Other commercially available chemicals and reagents were of analytical quality. Tg(Lyz: EGFP) adult zebrafish aged 3–4 months were sourced from the National Zebrafish Resource Center (Wuhan, China).

### 2.2 Screening and purification of OGP1-1

The OGP1-1 sample was isolated from oyster by a series of extraction and purification procedures, as previously described (C. Li et al., 2019). All operations were performed at 4 °C unless otherwise specified. A total of 1 kg of oyster visceral mass was extracted from 10 kg of the edible portion (fresh mass) of *O. gigas.* The oyster visceral mass (1 kg) was rinsed with deionized water and mashed with a tissue homogenizer for 2 min. Crude polypeptides were then extracted in 30 mM phosphate buffer (pH 8.0) using an ultrasonic processor (Xinzhi Co., Ningbo, China) equipped with a straight probe for continuous pulsing. Following centrifugation at 10,000 ×*g* for 30 min, the supernatant was precipitated with 70–100% saturation of ammonium sulfate. Following centrifugation at 10,000 ×*g* for 30 min, the precipitate was obtained and re-dissol*v*ed in 30 mM Tris-HCl buffer (pH 8.0), then dialyzed against deionized water and lyophilized. A total of 101.40 g of lyophilized polypeptide extract was obtained.

The lyophilized material (101.40 g) was implemented to an Agilent 1200 liquid chromatography system linked to a TSKgel G2000SWXL column (TOSOH Company, 7.8 × 300 mm, 10 μm, 125 A) for purification. The column was pre-equilibrated with a 50 mM sodium sulfate buffer at pH 8.0 and eluted at a flow rate of 0.5 mL/min. The detection wavelength was established at 280 nm, and the column temperature was maintained at 25 °C. Fractions were pooled, dialyzed, and lyophilized. The fractions designated P1 to P4 yielded 12.80 g, 4.60 g, 9.50 g, and 36.50 g, respectively. The fraction, P1, exhibiting the greatest anti-inflammatory activity, was subjected to an Ultimate LB-C8 column (Welch Company, 5 μm, 4.6 × 200 mm) attached to an Agilent 1200 liquid chromatography system for further purification. The elution solvent system consisted of water-trifluoroacetic acid (solvent A; 100:0.1, *v*/v) and acetonitrile-trifluoroacetic acid (solvent B; 100:0.1, v/v). A linear gradient of acetonitrile with 0.1% trifluoroacetic acid was applied from 10% to 50% over 50 min at a flow rate of 0.8 mL per minutes, resulting in the collection and lyophilization of the polypeptide OGP1-1. Ultimately, from 10.0 g of the P1 fraction, 625.00 mg of OGP1-1 was procured.

The anti-inflammatory efficacy of fractions was detected by a NO generation inhibitory activity assay. Briefly, RAW264.7 macrophage cells were initially seeded in 96-well microplates at a density of 1 × 10^6^ cells per milliliter and cultured until reaching 90% confluency. Three experimental groups including an untreated control, an LPS-treated group, and a sample group were created and treated with serum-free medium containing 1 μg/mL LPS alone or combined with 20 μM of fractions. After an 18-h incubation, NO production was measured spectrophotometrically at 540 nm using the Griess reaction system (Beyotime Biotechnology, Shanghai, China), following the manufacturer's standard protocols.

### 2.3 SDS-PAGE

OGP1-1 was analyzed by both reducing SDS-PAGE and non-reducing SDS-PAGE, employing an acrylamide concentration of 10% for the stacking gel and 15% for the running gel. Prior to analysis, OGP1-1 samples were co-incubated with non-reducing and reducing loading buffers in *v*olume ratios of 1:3 and 1:4, respectively. The mixtures were thereafter vortexed thoroughly and heated at 90 °C for 10 min. For electrophoresis, 5 μL of each prepared protein solution was loaded into the gel wells in conjunction with 5 μL of molecular weight standard. The separation procedure was conducted at a constant voltage of 75 *V* for a duration of 2 h. The gels were identified by the Coomassie blue staining technique.

### 2.4 Reversed-phase high-performance liquid chromatography

To ascertain the purity of OGP1-1, high-performance liquid chromatography (HPLC) examination was performed on an Agilent 1200 liquid chromatography system equipped with a ZORBAX®300SB-C8 column (4.6 × 150 mm, 5 μm, Agilent, CA, USA). The elution solvent system consisted of water-trifluoroacetic acid (solvent A; 100:0.1, *v*/v) and acetonitrile-trifluoroacetic acid (solvent B; 100:0.1, v/v). The elution parameters were 64% solvent A and 36% solvent B, a flow rate of 1 mL per minute, a detection wavelength of 280 nm, and a column temperature of 25 °C.

### 2.5 Protein determination and carbohydrate concentration assay

The protein concentration of OGP1-1 was determined by the Bradford assay, using BSA as the standard. The determination of protein concentration was achieved through spectrophotometric measurement at 595 nm. A calibration curve generated using specified amounts of BSA standards plotted against their matching A595 values, yielded a linear regression equation. The concentration of the purified polypeptide solution (0.1 mg/mL) was determined by applying its measured absorbance in accordance with the derived calibration equation.

The total neutral sugar content of OGP1-1 was determined by a modified colorimetric phenol‑sulfuric acid technique, with glucose serving as the standard. The absorbance at 490 nm was used to determine the carbohydrate content in the sample. The concentration of pure polypeptide was 0.5 mg/mL, and the standard curve was constructed using glucose standard solutions with varying concentrations on the abscissa and absorbance at 490 nm on the Y-axis. The regression equation was obtained. The concentration of sugar in the purified polypeptide was measured using its absorbance and the regression equation.

### 2.6 Molecular weight determination by mass spectrometry

The precise molecular weight of OGP1-1 was determined by electrospray ionization mass spectrometer (ESI-MS). Upon dissolving the polypeptide sample in ultrapure water, it is fed into the liquid phase system connected to the mass spectrometer, where ESI-MS is detected in positive ion mode, with a mass-nucleus ratio (*m*/*z*) scanning range of 500–3000. The original mass spectrum data of the sample is deconvolved by ProMass to determine the relative molecular mass.

### 2.7 UV–vis absorption spectroscopy

UV–vis absorption spectroscopy was performed on a UV-2450 UV–vis absorption spectrophotometer (Shimadzu, Osaka, Japan) fitted with a 1.0 cm quartz cell. The used wavelength range spanned from 190 to 400 nm.

### 2.8 Sequence identification of polypeptide OGP1-1

Polypeptide precipitation was initiated by mixing the sample with chilled acetone (5× volume) overnight at −20 °C. Following centrifugation, the pellet was subjected to three washes with ice-cold 90% acetone. The cleaned precipitate was resuspended in 8 M urea, augmented with protease inhibitors, incubated on ice for 30 min, and clarified by centrifugation. The supernatant was concentrated using ultrafiltration (8 M urea) to 50 μL, then diluted to 90 μL with lysis buffer, and sequentially alkylated with 10 mM TCEP (37 °C, 1 h) and 40 mM IAM (room temperature, 40 min, in the dark). Polypeptides were reprecipitated with acetone, pelleted, air-dried, and then reconstituted in 100 mM TEAB. Trypsin digestion (1:50 enzyme: substrate ratio, 37 °C, overnight) was conducted prior to vacuum drying.

Peptides were loaded onto an Eksigent 3C18-CL-1230 column (micro-LC 415 system) for LC-MS/MS, using mobile phases: A (2% ACN, 0.1% FA) and B (98% ACN, 0.1% FA). An 80- min gradient was implemented: 2–6% B (0–1 min), 6–20% B (1–65 min), 20–25% B (65–70 min), and 25–80% B (70–80 min) at 5 μL per minute. MS1 scans (*m*/*z* 350–1250, 0.25 s) and MS2 scans (m/z 100–1500, 0.05 s, high-sensitivity mode) were conducted in information dependent analysis (IDA) mode, prioritizing the top 20 precursors every cycle. Database searches against the *O. gigas* transcriptome ORF library (PEAKS Studio 8) included static cysteine carbamidomethylation, dynamic methionine oxidation/N-terminal acetylation, tryptic cleavage (≤2 missed sites), 20 ppm precursor mass tolerances, 0.1 Da fragment mass tolerances, and an FDR threshold of 1%.

### 2.9 Circular dichroism spectroscopy and Fourier transform infrared spectroscopy determination

An aqueous solution of OGP1-1 at a concentration of 20 μg/mL, prepared in PBS and distilled water, respectively, was sterile-filtered through a 0.02-μm pore-size membrane to exclude particle impurities. Spectral measurements were performed on a Jasco J-810 spectropolarimeter (JASCO Inc., Tokyo, Japan) equipped with a temperature-regulated sample chamber kept at 20 °C. Measurements employed 0.1 cm pathlength quartz cuvettes, with spectral data acquired in the far-UV region (190–250 nm). The instrumental parameters were a scanning rate of 50 nm per minute, a bandwidth of 2 nm, a data resolution of 0.2 nm, a sensitivity of 20 mdeg, and an integration time of 0.5 s. Each documented spectrum signifies the averaged signal from eight successive scans to enhance signal-to-noise ratios. Spectral data were subjected to background subtraction to eliminate solvent contributions and normalized to mean residue ellipticity (θ, mdeg·cm^2^·dmol^−1^). The composition of secondary structure, including α-helix, β-sheet, β-turn, and unordered coil fractions, was quantified through deconvolution algorithms implemented in the manufacturer's software package (JASCO Spectra Manager), utilizing reference datasets established by Yang (Yang et al., 1986). Measurements were conducted in triplicate.

OGP1-1, previously dissolved in PBS, was freeze-dried and combined with spectroscopic-grade KBr at a 1:100 (*w*/w) ratio, pulverized in a mortar, and compressed into a 1 mm thick disk. Spectra were obtained using a Fourier transform infrared spectrometer (Nicolet Apex FT-IR spectrometer, Thermo Fisher Scientific) in transmission mode by averaging 32 scans throughout a wavenumber range of 4000–400 cm^−1^.

### 2.10 Three-dimensional structure prediction of OGP1-1

The three-dimensional structure of OGP1-1 was computationally modeled by homology-based approaches utilizing AlphaFold web service. The amino acid sequence of OGP1-1 was submitted to generate the three-dimensional structure conformation. The resultant predicted model was subjected to energy minimization and stereochemical validation (pTM = 0.9), followed by structural visualization and analysis using the PyMOL system (Fig. S1).

### 2.11 Assessment of thermal and pH stability

The stability of OGP1-1 under varying thermal conditions and pH levels was evaluated based on a previously reported procedure (Yarnpakdee et al., 2015), with slight adaptations. For the thermal stability assay, polypeptide solutions with concentration of 10 μM were subjected to heating in a water bath at specified temperatures (25, 65, 75, 85, and 100 °C) for a duration of 30 min. The inhibitory effect of the heated samples on NO production was then evaluated in LPS-stimulated RAW264.7 macrophages. To analyze pH stability, peptide solutions were first adjusted to target pH values (3, 5, 7, 9, and 11) and maintained under ambient conditions for 60 min. Following readjustment of all samples to pH 7.0, the ability of the samples to inhibit NO production was measured according to the aforementioned protocol.

### 2.12 Quantitative PCR analysis in real time

RAW264.7 macrophages were seeded at a density of 1 × 10^6^/mL and randomly divided into three experimental groups: the control group cultured in RPMI1640 medium supplemented with 10% fetal bovine serum, the LPS model group treated with 1 μg/mL LPS, and the treatment group co-treated with 1 μg/mL LPS and OGP1-1 at 2.5, 5 or 10 μM. For all non-control groups, the cells were pre-incubated with LPS for 30 min prior to the addition of OGP1-1, and then the whole culture system was incubated for an additional 24 h. Total RNA was isolated from several cell groups using Trizol reagent for subsequent spectrophotometric quantification to evaluate gene expression. cDNA synthesis and amplification were performed with the One-Step PrimeScript RT-PCR Kit (Perfect Real Time, Takara, Japan), with sequence-specific primers for inflammatory markers (IL-6, TNF-α, IL-1β, IL-10) and the reference gene β-actin (Table S1). Following RNA isolation, samples were augmented with forward and reverse primers, and reactions were calibrated to 50 μL with RNase-free water. The thermal cycling conditions included an initial denaturation at 95 °C for 30 s, followed by 40 cycles of denaturation at 90 °C for 5 s and a simultaneous annealing/extension at 60 °C for 34 s. The relative quantification of target transcripts was calculated via the 2-ΔΔCt method, normalized against β-actin expression as an endogenous control (J. Li et al., 2024). A detailed MIQE (Minimum Information for Publication of Quantitative Real-Time PCR Experiments) checklist is provided in Supplementary Table S3.

### 2.13 ELISA experiment

RAW264.7 macrophages in the mid-logarithmic phase of development were prepared for enzyme-linked immunosorbent assay (ELISA). Cells were inoculated at a density of 1 × 10^6^/mL in 6-well plates, with 2 mL aliquots per well. Cultures were maintained under standard conditions (37 °C, 5% CO₂) until achieving confluency. Cells were randomly assigned to three experimental cohorts: control group (RPMI1640 medium with 10% fetal bovine serum), LPS model group (1 μg/mL LPS), and treatment group (LPS at 1 μg/mL co-administered with OGP1-1 at concentrations of 2.5, 5, or 10 μM). All non-control cultures received 30 min of pre-exposure to LPS before the introduction of OGP1-1, followed by a 24-h incubation period. The quantities of IL-1β, IL-6, and TNF-α in the supernatant were measured using commercial ELISA kits in accordance with the manufacturer's protocols.

### 2.14 NO release detection

RAW264.7 macrophage cells were initially plated onto 96-well microplates at a density of 1 × 10^6^ per milliliter and cultivated until reaching 90% confluency. Three experimental groups were subsequently established: an untreated group, an LPS-treated group, and a sample group treated with serum-free medium, 1 μg/mL LPS, 1 μg/mL LPS accompanied with 2.5, 5, or 10 μM of OGP1-1, respectively. A Following an 18-h incubation period, NO production was measured spectrophotometrically at 540 nm using the Griess reaction system (Beyotime Biotechnology, Shanghai, China), in strict accordance with the manufacturer's defined protocols.

### 2.15 Western blotting analysis

In Western blot investigations, cellular samples from all experimental cohorts were harvested and lysed for protein isolation. RAW264.7 macrophages were pretreated with OGP1-1 for 1 h, followed by stimulation with 1 μg/mL LPS for 24 h before harvesting. A quantitative evaluation of extracted proteins was performed through BCA test, followed by electrophoresis in a discontinuous system with 12% resolving and 5% stacking gels. Thereafter, resolved proteins were transferred to nitrocellulose membranes using a wet transfer method. Membranes were saturated for 2 h at ambient temperature with a blocking buffer containing 10% non-fat dried milk, followed by overnight incubation at 4 °C with incubated with primary antibodies (NF-κB p65, p-p65, IκBα, p-IκBα, TLR-4, β-actin). After three TBST rinses, species-matched HRP-conjugated secondary antibodies were incubated for 90 min at room temperature. Post-antibody treatment, membranes underwent consecutive TBST washes, and chemiluminescent signals were digitally captured as grayscale images. In semi-quantitative analysis, protein band intensities were adjusted to β-actin reference bands through densitometric evaluation.

### 2.16 LC-MS/MS based targets identification and pull-down determination

A click reaction mixture comprising 1 mM sodium ascorbate (NaVc), 100 mM THPTA, 1 mM CuSO_4_, and 50 μM TAMRA-azide was employed to conjugate a biotin tag to OGP1-1, yielding the OGP1-1 probe (OGP1-1-P). RAW 264.7 cells were categorized into three experimental conditions: a control (treated with PBS), a probe group (exposed to OGP1-1-P for 1.5 h), and a competition group (pre-treated with OGP1-1 for 3 h followed by OGP1-1-P for 1.5 h). Subsequently, the cells were lysed with PBS containing 0.1% Triton X-100. Proteins from all samples were precipitated using ice-cold acetone at −20 °C, redissolved in 0.1% SDS, and incubated with 50 μL of streptavidin-coated beads for 4 h. The beads were subjected to three sequential washes with 1% SDS, 6 M urea, and 1× PBS.

For on-bead digestion, captured proteins were reduced with dithiothreitol (DTT), alkylated using iodoacetamide (IAA), and subsequently digested overnight with trypsin at 37 °C. The resulting peptides were desalted using a commercial C18 column, chemically labeled via dimethylation, and finally subjected to LC-MS/MS analysis.

For pull down assay, after PBS washing, the beads were combined with 40 μL of 1× loading buffer and heated at 95 °C for 5 min to denature the bound proteins.

### 2.17 GO and KEGG enrichment analysis

Differentially expressed proteins were identified by comparing abundance profiles across the control (PBS-treated), probe (OGP1-1-P-treated), and competition (OGP1-1 + OGP1-1-P-treated) groups. Selection criteria included an absolute fold change (probe/compete) > 2 with an FDR-adjusted *p*-value <0.05, as well as an absolute fold change (probe/control) > 5 with FDR < 0.05 (Yu et al., 2012). Subsequently, the resulting protein set was subjected to Venn diagram analysis and Gene Ontology (GO) enrichment via the DAVID^+^ platform (Smedley et al., 2009).

### 2.18 Cellular thermal shift assay (CETSA)

CETSA experiment was carried out as reported previously (Hengst et al., 2020). Briefly, RAW 264.7 cell lysis were incubated with or without OGP1-1 for 3 h. Cell suspensions were then divided into eight PCR tubes and heated at the temperatures from 37 °C to 72 °C for 5 min by PCR analyzer. The lysates were then analyzed via western blot analysis.

### 2.19 Biomolecular interaction analysis

The recombinant human TAK1 protein with an N-terminal hexahistidine tag demonstrated purity exceeding 90%. In accordance with the Monolith NT Protein Labeling Kit RED-NHS (Nano Temper, Munich, Germany), the target protein was labeled with NT-647-NHS fluorescent dye. The OGP1-1 stock solution was formulated in 0.05% Tween 80/PBS, followed by dilution in PBS as per the guidelines of the Monolith NT.115 MicroScale Thermophoresis system. The labeled TAK1 was then included into the diluted OGP1-1 solution before the research began. Samples were loaded into Monolith NT.115 capillaries for examination using the MicroScale Thermophoresis device (Nano Temper, Germany). Subsequent signal capture and calculation of the dissociation constant were performed utilizing MO. Affinity Analysis software.

### 2.20 Molecular docking

AutoDock Vina is extensively employed in molecular docking investigations of ligand-receptor interactions owing to its computational efficiency and reliable predictive performance. Among available approaches, semi-flexible docking proves particularly suitable for modeling biomolecular recognition processes (Zhu et al., 2022). Accordingly, this study implemented a semi-flexible docking simulation between OGP1-1 and TAK1 utilizing AutoDock 4.2 (Scripps Research Institute, La Jolla, CA, USA). The crystallographic structure of the TAK1 chimera was retrieved from the RCSB Protein Data Bank (accession code: 5V5N), and the three-dimensional model of OGP1-1 was generated using AlphaFold 3 as described above. Preprocessing of the initial structures was conducted with AutoDock Tools 1.5.6, which involved elimination of non-polar water molecules, incorporation of polar hydrogens, and preservation of native atomic charges for both TAK1 and OGP1-1, followed by export in .pdbqt format. A docking grid was centered at the original ligand's binding site (coordinates: x = 12.05, y = −3.86, z = 16.25) with dimensions of 60 × 60 × 60 Å^3^ and a grid point spacing of 0.60 Å. Docking simulations were executed over 100 independent iterations using the Lamarckian genetic algorithm under default parameter configurations. To validate the reliability of the docking protocol, a redocking experiment was performed using the known TAK1 inhibitor takinib (Totzke et al., 2017). The crystallographic structure of TAK1 (PDB: 5V5N) contains no bound small-molecule ligand; therefore, takinib was docked into the same binding pocket using the identical grid parameters. The resulting docking pose reproduced the reported binding mode with a root-mean-square deviation (RMSD) of 1.2 Å, confirming that the docking parameters are appropriate for predicting ligand–TAK1 interactions. The complex conformation exhibiting the most favorable binding energy was chosen for subsequent structural visualization in PyMOL. Predicted binding modes were further examined for intermolecular contacts using LigPlot+.

### 2.21 Molecular dynamics (MD) simulations and trajectory analysis

The MD simulations of selected TAK1-polypeptide complexes were performed over a 100 ns period using Gromacs 2022.3 software (Van Der Spoel et al., 2005). Preprocessing of the polypeptide involved assigning the GAFF force field via AmberTools22. Hydrogenation and RESP charge calculations were conducted with Gaussian 16 W. The resulting potential parameters were incorporated into the system topology. Simulations were run at a constant temperature of 300 K and a pressure of 1 bar. The Amber99sb-ildn force field was applied, with the TIP3P water model serving as the solvent. System neutrality was achieved through the addition of Na^+^ ions. Energy minimization was conducted using the steepest descent algorithm. This was followed by equilibration in the isothermal isovolumic ensemble (NVT) and isothermal isobaric ensemble (NPT), each for 100 ps with a coupling constant of 0.1 ps. Production MD was then executed for 100 ns, using a 2 fs integration step over 5 million iterations. Trajectory analysis was performed using built-in Gromacs utilities to compute the root-mean-square variance (RMSD), root-mean-square fluctuation (RMSF), radius of gyration (Rg) values of each amino acid trajectory, and solvent accessible surface area (SASA). These metrics were integrated with binding free energy estimates from MMGBSA and free energy landscape analyses.

### 2.22 Immunofluorescent assay

Cultured cell slides representing each experimental group were subjected to three consecutive washes with PBS. Fixation was then conducted using 4% paraformaldehyde for a 15-min incubation, followed by three rinses with PBS. Endogenous peroxidase activity was inhibited by hydrogen peroxide treatment, subsequently followed by serum-based blocking. Primary antibodies targeting NF-κB p65 were applied for 1-h incubation at 37 °C, followed by washing cycles with PBS. Fluorescent-conjugated secondary antibodies were administered by dropwise application in ambient darkness conditions. Post-triplicate PBS washes, nuclear visualization was accomplished using DAPI staining, supplemented by anti-photobleaching mounting medium application (Y. Li, Xu, et al., 2024). The localization patterns of NF-κB p65 were eventually elucidated using laser confocal microscopy imaging.

### 2.23 Anti-inflammatory effects of OGP1-1 in a zebrafish inflammation model

Zebrafish were maintained in a controlled aquatic facility, with distinct strains and sexes housed separately. The daily provision of live brine shrimp and commercial pellet feed ensured nutritional requirements. The environmental conditions consisted of a water temperature of 28.0 ± 0.5 °C, maintained by an automated climate-control system, and a photoperiod of 14 h of light and 10 h of darkness. For breeding, sexually mature males and females were relocated to spawning tanks at ratios of 1:1 or 1:2, separated to prevent premature interaction. Fertilized eggs were harvested for one-hour post-partition removal, rinsed in sterile zebrafish medium, and incubated under light at 28 °C. Phenylthiourea was administered 6 h post-fertilization (hpf) to inhibit melanogenesis, maintaining optical clarity for further imaging. All animal procedures were carried out in accordance with the EU Directive 2010/63/EU for the protection of animals used for scientific purposes and the NIH Guide for the Care and Use of Laboratory Animals. The experimental protocol was approved by the Experimental Animal Ethics Committee of Qilu Hospital of Shandong University (KYLL-2025(ZM)-634).

Transgenic zebrafish embryos exhibiting macrophage-specific Lyz: EGFP fluorescence at 72 hpf were identified via stereomicroscopy. Ten larvae were distributed per well on 24-well plates for treatment. The experimental groups included: (1) untreated controls (fresh culture medium), (2) a positive control (20 μM ibuprofen), and (3) test groups (2.5, 5, or 10 μM samples). Following a 6-h pretreatment, all groups except controls were exposed to 20 μM CuSO_4_ in darkness for 1 h. Post-exposure, larvae underwent three washes with culture medium and were then sedated with 0.25% tricaine for fluorescence imaging. Macrophage populations in the caudal region were quantified using Image-Pro Plus software.

For qPCR analysis, juvenile zebrafish (*n* = 50 per group) were harvested in 1.5 mL tubes, rinsed thrice with distilled water, flash-frozen in liquid nitrogen, and preserved at −80 °C for 48 h. Total RNA was extracted using Trizol reagent, followed by cDNA synthesis employing the One-Step PrimeScript RT-PCR Kit. Quantitative PCR amplification was performed with the following thermal cycling parameters: initial denaturation at 95 °C for 5 min, followed by 40 cycles of denaturation at 95 °C for 10 s and simultaneous annealing/extension at 55 °C for 30 s. Fluorescence signals were automatically captured after each cycle. The relative quantification of target transcripts was determined using the 2-ΔΔCt method, with β-actin serving as an endogenous control.

### 2.24 Statistical analysis

The experimental data were presented as means ± standard deviation (in vitro) or standard error of the mean (in vivo). Graphpad prism 9.5 (GraphPad Software, San Diego, USA) was used for statistical analysis. Comparisons between the two groups were conducted using the *t*-test, while multiple comparisons were assessed by One-way analysis of variance (ANOVA), with *P* < 0.05 being statistically significant.

## 3 Results and discussion

### 3.1 Screening and purification of anti-inflammatory polypeptide from oyster visceral mass

**Fig. 1:**
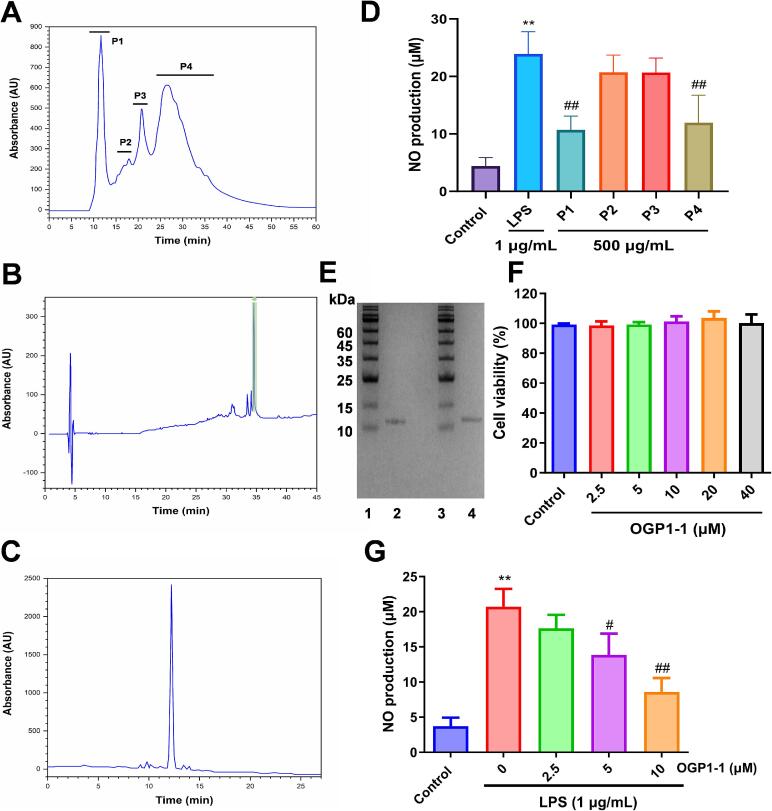
Purification and identification of polypeptide OGP1-1 from *O. gigas*. (A) Isolation of *O. gigas* polypeptide extract by a TSKgel G2000SWXL column. (B) Isolation of native OGP1-1 (green arrow) from the pooled polypeptide fraction by a C18 RP-HPLC column. (C) The purity profile of OGP1-1 detected by RP-HPLC. (D) Inhibition of NO production in LPS-stimulated RAW264.7 macrophages induced by P1-P4 fractions. Three independent experiments were conducted to calculate the mean ± SD (**P < 0.01 vs. the control group; #*P* < 0.05, ##*P* < 0.01 vs. LPS group). (E) Reducing and non-reducing SDS-PAGE profiles of OGP1-1, respectively. Lane 1, Marker, Lane 2, OGP1-1 reducing sample, Lane 3, Marker, Lane 4, OGP1-1 non-reducing sample. (F) Evaluation of cell viability of OGP1-1 in LPS-stimulated RAW264.7 macrophages. (G) Inhibition of NO production in LPS-stimulated RAW264.7 macrophages induced by OGP1-1. Three independent experiments were conducted to calculate the mean ± SD (**P < 0.01 vs. the control group; #*P* < 0.05, ##*P* < 0.01 vs. LPS group). (For interpretation of the references to colour in this figure legend, the reader is referred to the web version of this article.)

Polypeptides obtained from marine organisms represent a valuable resource in nutritional science, with contemporary research in protein chemistry emphasizing their diverse biological activities and therapeutic potential. *O. gigas* is a famous seafood around the world as well as a traditional marine Chinese medicine that has shown good efficacy in anti-inflammatory therapy, as per historical records in China. This work included screening for anti-inflammatory constituents in the polypeptide extract of *O. gigas*. A polypeptide, identified as OGP1-1, was isolated using a sequential purification procedure, including ammonium sulfate fractionation, size-exclusion, and reversed-phase high-performance liquid chromatography (Fig. 1A & B). NO inhibition was seen in RAW264.7 cells during the in vitro anti-inflammatory screening (Fig. 1D). The ultimate production of OGP1-1 from the consumable fraction (fresh mass) of *O. gigas* was 0.00625%. The protein content of OGP1-1 was 99.0% (*w*/w), as determined by the Bradford method. Carbohydrate analysis verified the absence of glycosylation, aligning with its designation as a non-glycopeptide. RP-HPLC analysis confirmed homogeneity, exhibiting a singular symmetrical peak (Fig. 1C). The findings demonstrated that OGP1-1 is probably a native and pure non-glycopeptide.

SDS-PAGE is an essential method for the separation and analysis of proteins based on their molecular weights. The subunit composition of OGP1-1 was determined by reducing SDS-PAGE and non-reducing SDS-PAGE (Fig. 1E). SDS-PAGE analysis revealed that OGP1-1 is a monomeric polypeptide with an approximate mass of 12 kDa. Electrophoretic profiles revealed no detectable impurities, with a consistent band indicating compositional homogeneity. The results demonstrated that OGP1-1 is a monomeric, homogeneous polypeptide post-purification.

We further evaluated the cytocompatibility and anti-inflammatory potential of OGP1-1 in LPS-activated RAW264.7 murine macrophage cells. As demonstrated in Fig. 1F, OGP1-1 exhibited a nontoxic profile at all evaluated doses, hence confirming its appropriateness for following investigations.

NO, a principal inflammatory mediator excessively generated by iNOS after LPS stimulation, contributes to pathological inflammation and endotoxemia (Abulaiti et al., 2024). Inhibition of iNOS-induced NO overproduction is a therapeutic approach for mitigating inflammatory cascades. Macrophages were pretreated with different dosages of OGP1-1 before exposure to LPS, followed by the measurement of NO. Untreated cells exhibited basal NO levels of 3.73 μM, which escalated to 20.72 μM post-LPS stimulation, indicating a pronounced inflammatory activation in RAW264.7 cells. OGP1-1 pretreatment significantly reduced LPS-induced NO increase in a dose-dependent manner (Fig. 1G, *P* < 0.01). These findings align with previous studies demonstrating that seafood-derived bioactive polypeptides inhibit iNOS-driven NO synthesis in LPS-stimulated macrophages (Kim et al., 2016; Shahidi & Saeid, 2025). Specifically, several anti-inflammatory peptides derived from salmon byproduct hydrolysates (Ahn et al., 2012; Ahn et al., 2015) and pearl oyster (*Pinctada martensii*) (Shen et al., 2023) have been reported to suppress NO and pro-inflammatory cytokine production. Compared to these short-chain oligopeptides (molecular weight < 1 kDa), OGP1-1 is a larger (11.95 kDa) polypeptide with a defined cupin domain, suggesting a distinct structural basis for its bioactivity.

### 3.2 Structural characterization of OGP1-1

**Fig. 2:**
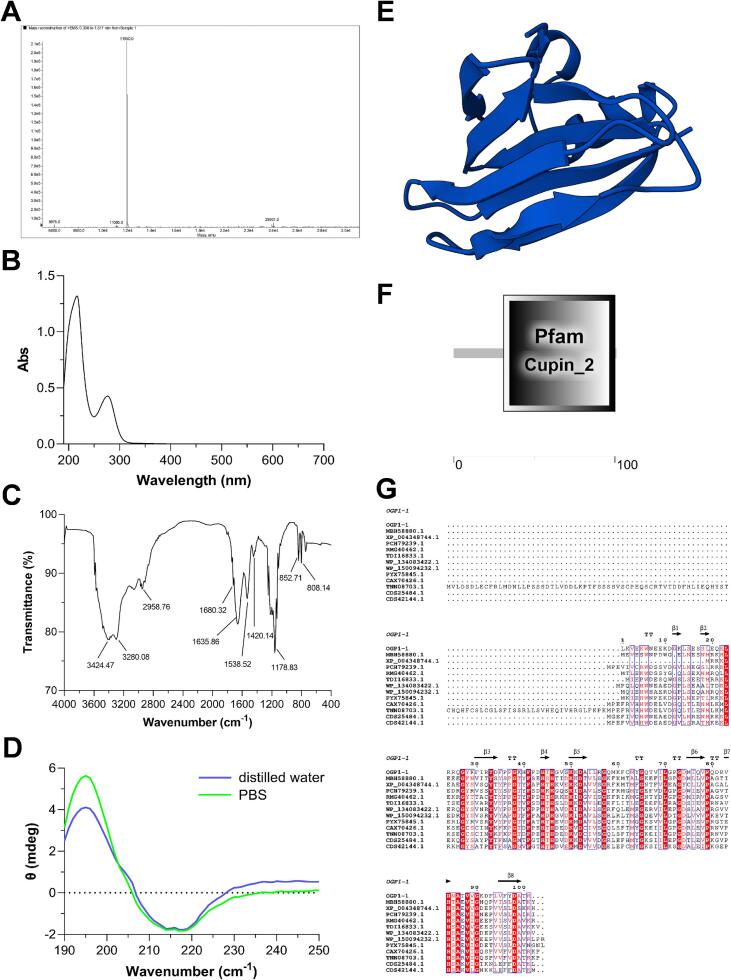
Structure characterization and physicochemical property analysis of OGP1-1. (A) The molecular weight of OGP1-1 determined by ESI-MS. (B) UV–vis spectrum of OGP1-1. (C) FT-IR spectrum of OGP1-1 in PBS and distilled water, respectively. (D) Secondary structure determination of OGP1-1 by circular dichroism spectra in PBS and distilled water, respectively. (E) Three-dimensional predicted structure of OGP1-1 using AlphaFold modeling. (F) Protein domain prediction of OGP1-1 through SMART server. (G) Sequence alignment between OGP1-1 and other polypeptides of cupin superfamily. Identical amino acid residues were in the red background. (For interpretation of the references to colour in this figure legend, the reader is referred to the web version of this article.)

Mass spectrometry is an effective instrument in polypeptide research for identification, quantification, and analysis. The precise molecular weight was measured by ESI mass spectrometry as 11.950 kDa (Fig. 2A), corroborating the findings from SDS-PAGE. Prior studies have examined several anti-inflammatory peptides with a molecular weight of less than <1 kDa, derived from oyster hydrolysates (Ngandjui, Kereeditse, Kamika, Madikizela, & Msagati, 2024; Shahidi et al., 2025). Three peptides, TWP (402.19 Da), TAMY (484.19 Da), and FPGA (390.19 Da), derived from pearl oyster (*Pinctada martensii*) flesh hydrolysates, exhibited anti-inflammatory properties (Shahidi et al., 2025). In contrast to low molecular weight peptides that resemble small molecules, polypeptides have more complex spatial structures (L. Wang et al., 2022). This facilitates stable three-dimensional conformations that enhance binding to inflammatory receptors. Furthermore, polypeptides exhibited greater bioactivity at less dosages and are less vulnerable to rapid proteolytic degradation (Apostolopoulos et al., 2021).

The physicochemical characteristics of OGP1-1 were further evaluated. Fig. 2B illustrates the UV–vis absorption spectrum of OGP1-1. The spectrum exhibits strong absorption peaks at around 200 nm and a shoulder peak at 280 nm. These represent the characteristic absorption of the polypeptide backbone structure and the aromatic amino acid residues of OGP1-1.

FT-IR spectroscopy is an effective method for elucidating the structure and conformational dynamics of polypeptides. The amide I bands were identified in the FT-IR spectra at 1635.86 cm^−1^ and a less intense band at 1680.32 cm^−1^, as shown in Fig. 2C. A relatively intense band indicative of amide II was seen with an absorption frequency of 1538.52 cm^−1^. The hydroxyl stretching vibration accounts for the broad band in the spectrum ranging from 3280.08 to 3424.47 cm^−1^. The amide I band, situated between 1600 and 1700 cm^−1^ (C=O stretch) and the amide II band, approximately at 1550 cm^−1^ (C=N stretch coupled with the N—H bending form), indicate that the absorption distribution of amide I and amide II in OGP1-1 aligns with the typical bands for polypeptides (Lorenz-Fonfria, 2020). Furthermore, the β-sheet band frequency and the high-frequency vibration of the anti-parallel β-sheet structure are commonly recognized as absorption band frequencies at roughly 1680 and 1630 cm^−1^, respectively (Lorenz-Fonfria, 2020). The notable presence of β-sheet secondary structure in OGP1-1 is shown by the characteristic absorptions at 1680.32 and 1635.86 cm^−1^. A recent study demonstrated that polypeptides having a β-sheet secondary conformation exerted better anti-inflammatory activity (Cho et al., 2025).

The secondary structure of polypeptides may be rapidly determined via circular dichroism (CD) spectroscopy. Fig. 2D illustrates that the extensive negative ellipticity signal at 218 nm and the pronounced positive peak at 195 nm in the OGP1-1 CD spectra are characteristic of polypeptide β-sheet structure. The acquired CD spectrum, analyzed using the Jasco secondary structure estimation program, indicated that the secondary structure composition of OGP1-1 comprises 50.1% β-sheets, 6.9% α-helices, 9.0% β-turns, and 34.0% random coils. More than 65% of the secondary structure included the aggregate content of α-helix, β-sheet, and β-turn. OGP1-1 seems to be a polypeptide characterized by a stable and highly organized structure. Biological macromolecules especially polypeptides are present in ambient conditions in living organisms where external medium plays key role in protein functionality and stability. PBS is one of the acid-base buffer systems of extracellular fluids of organisms. We subsequently measured the CD spectrum of OGP1-1 in PBS. The spectrum exhibited two characteristic negative peaks at approximately 208 nm and 222 nm, consistent with the CD profile obtained in deionized water (Fig. 2D). This result parallels that of the anti-inflammatory peptide AC12 derived from the epidermal secretion of *Hypsiboas raniceps*, which exhibited β-sheet-turn-β-sheet structure and demonstrated effectiveness in reducing inflammatory indicators like IL-12, TNF-α, and NO (Popov et al., 2019). Accumulating evidence supports that a high β-sheet content contributes to the anti-inflammatory activity of bioactive peptides. For instance, a β-sheet-rich peptide from oyster hydrolysates exhibited potent inhibition of LPS-induced inflammation (Cho et al., 2025). Similarly, synthetic β-sheet peptides have been shown to interfere with TLR4 signaling, thereby reducing cytokine release. These observations suggest that the predominant β-sheet conformation of OGP1-1 may be critical for its stable interaction with TAK1 and subsequent anti-inflammatory effects.

Furthermore, tandem mass spectrometry (MS/MS) was employed to ascertain the whole amino acid sequence of OGP1-1. Following enzymatic digestion with trypsin, the resultant peptide fragments were examined and compared with the oyster transcriptome dataset (Table S2). Sequence analysis indicated that OGP1-1 has 101 residues, with a predicted isoelectric point of 9.27. The three-dimensional conformation of OGP1-1 was anticipated using AlphaFold modeling (Fig. 2E). Functional domain annotation further identified a signature cupin domain, characterized by a conserved β8-barrel fold, inside OGP1-1, therefore affirming its membership within the cupin superfamily (Fig. 2F).

Sequence homology evaluation was performed via the NCBI platform utilizing the BLASTp algorithm. The uncharacterized protein LOC128156200 from the Oyster *Ostrea angulata* was determined to be identical to OGP1-1. OGP1-1 demonstrated some similarity with polypeptides from the cupin superfamily, as illustrated in Fig. 2G. Additionally, the result demonstrated the constant distribution of secondary structure among these polypeptides. OGP1-1 could be identified as a cupin-like polypeptide.

### 3.3 Effect of OGP1-1 on pro-inflammatory cytokines production in LPS-stimulated RAW264.7 macrophages

**Fig. 3:**
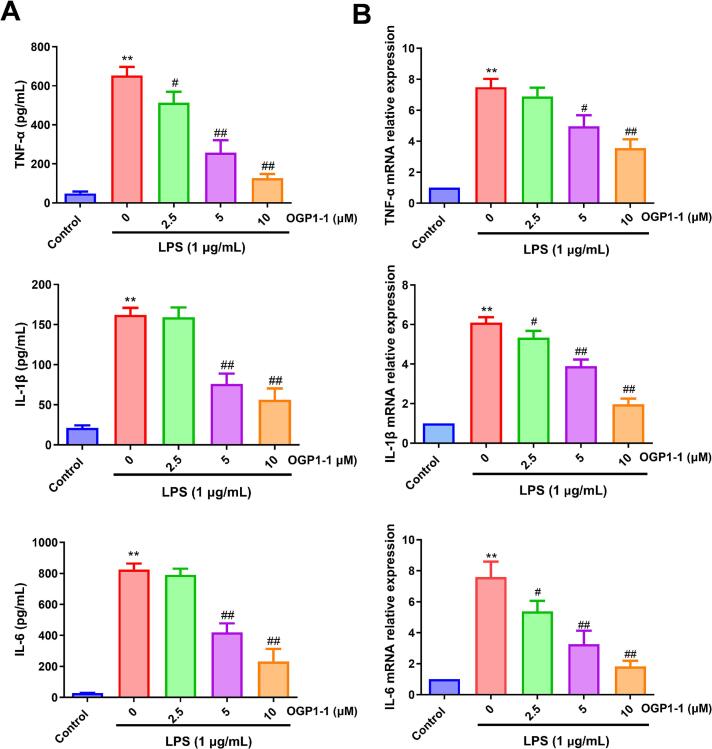
Effect of OGP1-1 treatment on the expression and production of proinflammatory cytokines in LPS-stimulated RAW264.7 macrophages. (A) Levels of TNF-α, IL-6, and IL-1β cytokines secreted by LPS-activated RAW264.7 macrophages, determined by ELISA. (B) mRNA levels of TNF-α, IL-6, and IL-1β in LPS-induced RAW264.7 macrophages, detected by qRT-PCR. Three independent experiments were conducted to calculate the mean ± SD (**P < 0.01 vs. the control group; #*P* < 0.05, ##*P* < 0.01 vs. LPS group).

Elevated expression of inflammatory mediators, particularly proinflammatory cytokines, has been associated with the maintenance of chronic inflammatory conditions, a central driver of multiple human pathologies (Y. Liu et al., 2025). Given this pathogenic association, prioritizing the reduction of proinflammatory cytokine production is essential for preventing chronic inflammation. We evaluated the inhibitory effects of OGP1-1 on pro-inflammatory cytokines using a qPCR or ELISA assay. As shown in Fig. 3, LPS-stimulated RAW264.7 macrophages exhibited significantly elevated secretory levels of IL-6, TNF-α, and IL-1β in comparison to untreated controls (*P* < 0.01). Strikingly, pretreatment with 5 and 10 μM OGP1-1 induced a dose-dependent reduction of these cytokines (*P* < 0.01), with IL-6 exhibiting the highest sensitivity to inhibition. These results validate that OGP1-1 effectively inhibited the secretion of proinflammatory cytokines, underscoring its potential for ameliorating inflammatory cascades.

### 3.4 Effect of OGP1-1 on iNOS/COX-2 protein expressions

**Fig. 4:**
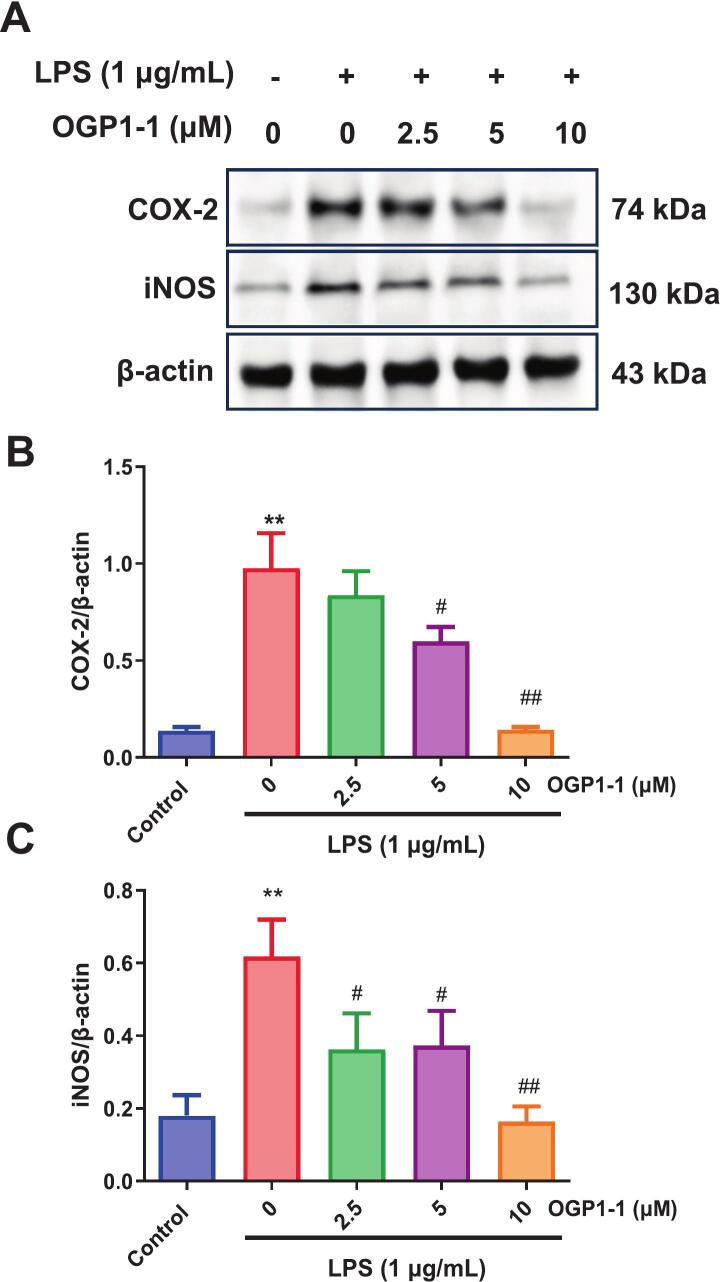
Effects of OGP1-1 treatment on iNOS and COX-2 protein expression in LPS-stimulated RAW264.7 macrophages. (A) Protein levels measured by western blotting analysis; (B) relative levels of COX-2 protein; (C) relative levels of iNOS protein. Three independent experiments were conducted to calculate the mean ± SD (**P < 0.01 vs. the control group; #*P* < 0.05, ##*P* < 0.01 vs. LPS group).

The overexpression of iNOS and COX-2 has been identified as a pivotal factor in chronic inflammatory disorders. Under inflammatory conditions, these enzymes facilitate the overproduction of NO and prostaglandin E2 (PGE2), amplifying tissue damage and systemic inflammation (X. Liu et al., 2024). Various stimuli, including proinflammatory cytokines and bacterial LPS, may regulate the synthesis of iNOS and COX-2 by activating the transcription factor NF-κB (Q. Li & Verma, 2002). To ascertain whether OGP1-1 inhibited the production of NO and proinflammatory cytokines, the protein expressions of COX-2 and iNOS were measured by western blot analysis in RAW264.7 macrophages. As illustrated in Fig. 4, LPS stimulation significantly enhanced the protein levels of iNOS and COX-2 relative to untreated controls, hence validating the activation of the inflammatory system. Notably, OGP1-1 pretreatment significantly suppressed LPS-induced protein overexpression, with almost total inhibition observed at 10 μM for both iNOS and COX-2 (Fig. 4B, C).

### 3.5 Thermal and pH stability

The inhibitory capacity of OGP1-1 against NO production remained largely unaffected by thermal processing. As illustrated in Fig. S2A, samples exposed to temperatures ranging from 25 to 100 °C exhibited NO concentrations between 8.62 and 12.06 μM, with no statistically significant variation across the different treatment groups. Similarly, the peptide maintained its functional activity across a broad pH spectrum (pH 3–11), yielding measured NO levels from 9.55 to 12.18 μM (Fig. S2B), and no significant differences were detected among the various pH conditions. Given that variations in temperature and pH are common during food processing and storage, the retention of bioactivity under such stresses is a key functional attribute. The data confirm that OGP1-1 effectively preserves its NO-inhibitory activity following exposure to both heat and diverse pH environments.

### 3.6 Anti-inflammatory effect of OGP1-1 in zebrafish model

**Fig. 5:**
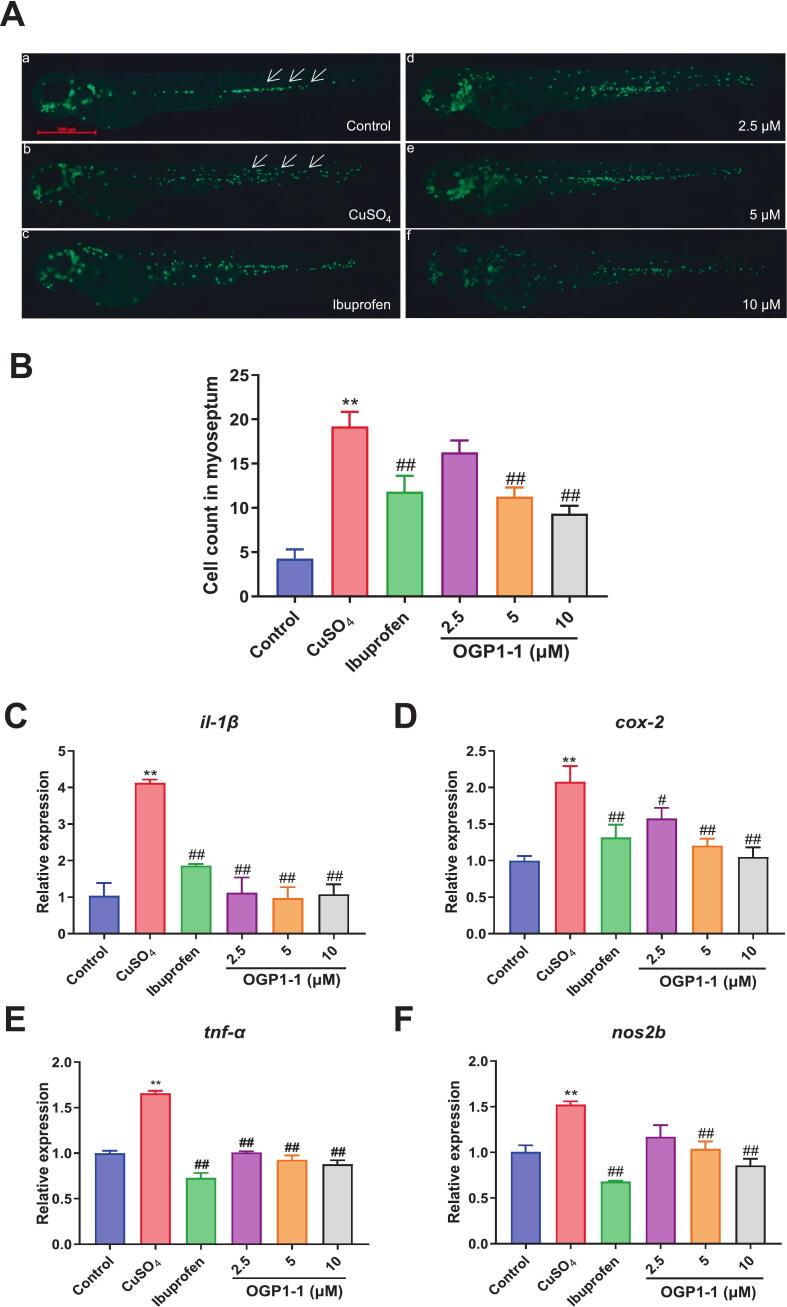
Effect of OGP1-1 on CuSO_4_-induced inflammation in zebrafish model. (A) Representative green-fluorescent neutrophils from different groups (bar = 200 μm). The white arrow indicated the inflammatory cells migrate from circulation to injured lateral line nervous. (B) Quantitative analysis of the number of macrophages in the zebrafish inflammatory models before and after the administration of OGP1-1 and ibuprofen, respectively. Data were expressed as mean ± SEM; **P < 0.01 vs. Control group; ## *P* < 0.01 vs. CuSO_4_ group, *n* = 10 larvae each group. The effects of OGP1-1 on the expression levels of inflammation-related genes, *il-1β* (C), *cox-2* (D), *tnf-α* (E), and *nos2b* (F), in zebrafish. Gene mRNA expression is shown as the relative expression by fold compared to the control. Data were expressed as mean ± SD; **P < 0.01 vs. Control group; ## *P* < 0.01 vs. CuSO_4_ group, *n* = 50 juvenile zebrafish per group. (For interpretation of the references to colour in this figure legend, the reader is referred to the web version of this article.)

We used zebrafish in in vivo investigations to further corroborate the anti-inflammatory effects of OGP1-1. Zebrafish, a prominent animal model characterized by rapid reproduction, genome similarity, and in vivo imaging capabilities, has been extensively used in inflammation research. As illustrated in Fig. 5A, B, the migratory rate of immune cells in the model group of zebrafish was significantly elevated (*P* < 0.01) compared to the blank control group, indicating that exposure to CuSO_4_ solution elicited an inflammatory response (Olivari et al., 2008). In comparison to the model group, the ibuprofen plus OGP1-1 therapy resulted in a substantially reduced number of migrating zebrafish immune cells (*P* < 0.01). Moreover, the quantity of immune cells in the OGP1-1 treatment groups decreased in a dose-dependent manner, indicating that OGP1-1 exhibited an anti-inflammatory effect.

### 3.7 Effect of OGP1-1 on the expression levels of inflammation-related genes in zebrafish

To further validate the in vitro findings, we assessed the expression levels of pro-inflammatory genes in zebrafish through a qPCR assay. As illustrated in Fig. 5C-F, CuSO_4_ significantly increased the expression levels of *il-1β*, *cox-2*, *tnf-α*, and *nos2b* in the model group relative to the blank control group (*P* < 0.01); however, OGP1-1 effectively inhibited the upregulation of these genes (*P* < 0.01). Zebrafish-specific antibodies are extremely uncommon relative to other animal models (Gut et al., 2017). Considering the physiological and metabolic disparities between zebrafish and mammals, future studies will utilize various animal models, such as mice, to further investigate the anti-inflammatory mechanisms of OGP1-1 and ascertain its target protein.

### 3.8 OGP1-1 directly targets TAK1 in LPS-induced RAW264.7 cells detected by chemical proteomics

**Fig. 6:**
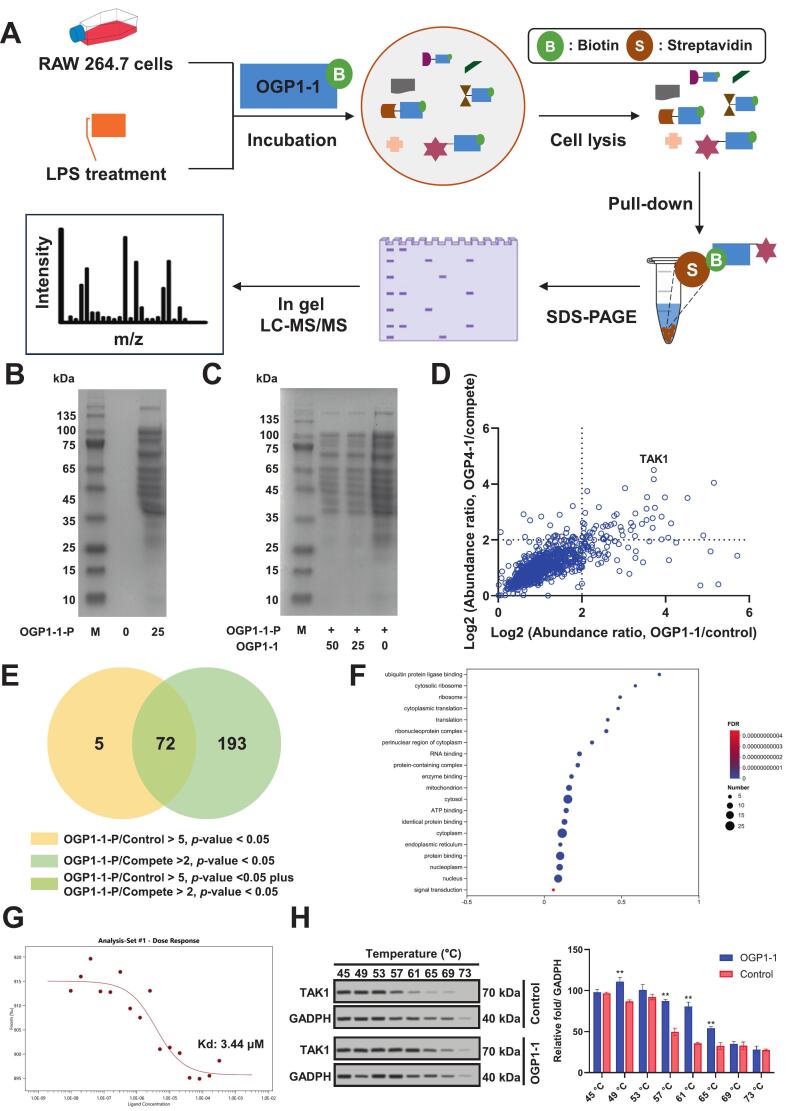
Chemical proteomics identified the binding targets of OGP1-1 in RAW 264.7 cells. (A) General workflow for Chemical proteomics profiling of OGP1-1 targets. (B) Labelling for OGP1-1 probe (OGP1-1-P) in RAW 264.7 cells. (C) The competition of proteins labeled with OGP1-1-P by excess OGP1-1. (D) Scatter plot depicting the differential enrichment of proteins. TAK1 is the potential target (Top1 in fold change of OGP1-1-P/Compete). (E) Venn digraph displaying the amount and overlap of proteins both enriched in those two groups. (F) Scatter diagram for gene ontology enrichment analysis of differential proteins. (G) The binding instant (Kd) between OGP1-1 and TAK1 determined by MST. (H) CETSA-WB and statistical analysis of the protein levels, Data were expressed as mean ± SD; **P < 0.01 vs. Control group.

To identify potential molecular targets of OGP1-1, we employed a chemical proteomics strategy, as outlined in Fig. 6A. RAW 264.7 cells stimulated with LPS were incubated with increasing concentrations of a biotin-conjugated OGP1-1 (OGP1-1-P). The cells were subsequently lysed and subjected to streptavidin bead pulldown via non-covalent affinity binding. Proteins captured by OGP1-1-P were resolved using SDS-PAGE. Control experiments were included to confirm band specificity. The labeling profiles of OGP1-1-P are presented in Fig. 6B.

We further evaluated the competitive binding between OGP1-1 and OGP1-1-P in situ. Pre-treatment with unmodified OGP1-1 led to a concentration-dependent reduction in OGP1-1-P labeling intensity, with near-complete loss of signal at 50 μM competitor (Fig. 6C). These results indicate that OGP1-1-P specifically engages with native cellular targets of OGP1-1.

To characterize the captured proteins, affinity-enriched samples were digested and analyzed by LC-MS/MS following established procedures (C. Li et al., 2024). Peptide spectra were matched to protein identities, and high-confidence candidates were visualized using scatter plots (Fig. 6D). Selection criteria included a fold change ≥2 for probe/competition and ≥ 5 for probe/control, yielding 77 and 265 potential targets, respectively. Intersection of these sets identified 72 common proteins (Fig. 6E). Gene Ontology analysis indicated significant enrichment in protein binding and protein processing in cytoplasm and nucleuplasm (Fig. 6F).

Among the candidate interactors, transforming growth factor-β-activated kinase 1 (TAK1) was prioritized due to its top-ranking position based on competitive binding ratio. TAK1 is a central regulator in NF-κB and MAPK signaling, activating downstream pathways such as JNK and p38, and is implicated in stress response, apoptosis, and inflammatory processes (Xu et al., 2020). Elevated TAK1 expression has been associated with pathological conditions including liver fibrosis, nonalcoholic steatohepatitis, and cardiovascular damage (Dai et al., 2022; W. Wang et al., 2021). We therefore speculated that TAK1 might be the key target protein of OGP1-1.

Furthermore, we validated this interaction using microscale thermophoresis (MST), which confirmed direct binding with a dissociation constant (Kd) of 3.44 μM (Fig. 6G). Further support came from a cellular thermal shift assay (CETSA), where OGP1-1 treatment significantly enhanced the thermal stability of TAK1 in cell lysates (Fig. 6H), consistent with target engagement.

### 3.9 Molecular docking and molecular dynamics simulations analysis for binding of OGP1-1 to TAK1

**Fig. 7:**
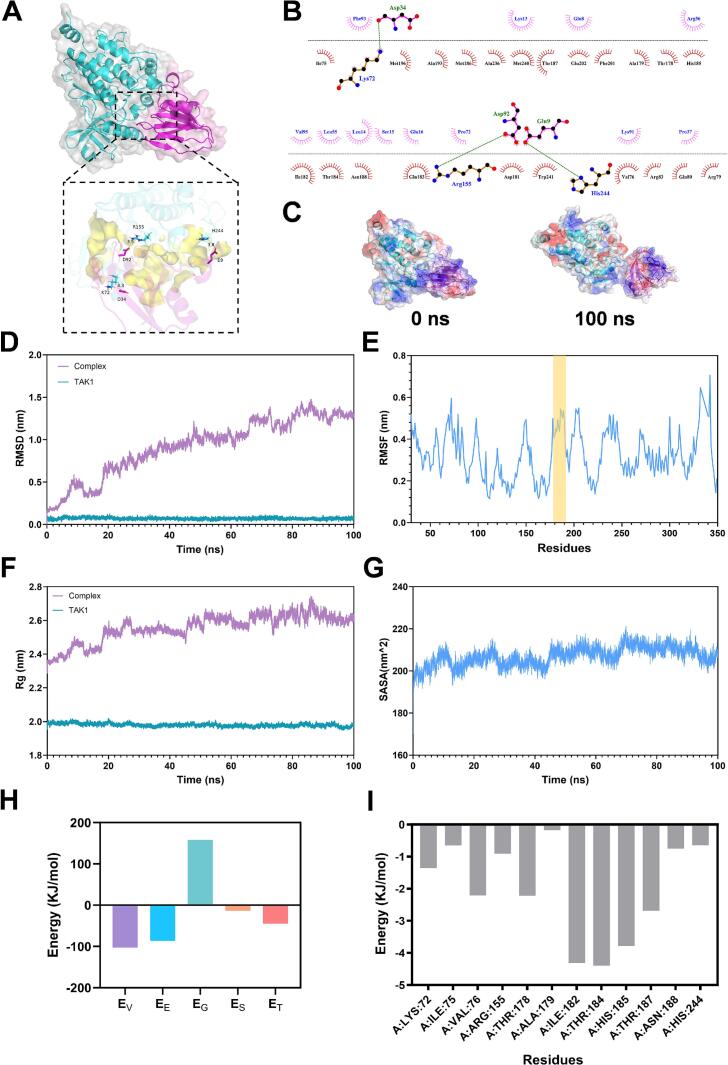
Analysis of molecular dock and molecular dynamics simulations of OGP1-1 binding to TAK1 (PDB 5V5N). (A) Molecular docking of OGP1-1 (purple) and TAK1 (cyan). The yellow spheres represent the hydrophobic interaction interface. (B) Binding modes of OGP1-1 with TAK1. Green dot lines represent hydrogen bond. Red eyelash curves represent hydrophobic interaction. (C) Conformation of OGP1-1 (purple) at 0 ns and 100 ns in MD simulations. (D) RMSD fluctuations of TAK1 backbone atoms and complex during the 100 ns MD simulation. (E) RMSF of TAK1 Cα atoms. (F) Rg plot. (G) Average SASA plot. (H) Binding free energy calculated using MM-GBSA analysis of the final 100 ns MD simulation. (I) Residue energy contribution decomposition based on the final 100 ns MD simulation. Abbreviations: Et: total binding energy; Ev: van der Waal energy; Ee: electrostatic energy; Es: Solvent accessible surface area energy; Ep: Polar solvation energy. (For interpretation of the references to colour in this figure legend, the reader is referred to the web version of this article.)

Molecular docking serves as a computational method to predict atomic-level interactions between ligands, including polypeptides or small molecules and receptor proteins. Through simulation of the binding process, it facilitates the identification of optimal binding conformations and interaction strengths, typically quantified by binding scores or free energy values. This approach offers critical insights into structural and functional characteristics of molecular recognition events, rendering it a fundamental technique in biomolecular studies, pharmaceutical development, and peptide design (Aguiar et al., 2025; Hou et al., 2025). In light of the marked anti-inflammatory properties and TAK1-binding ability of OGP1-1, molecular docking was performed targeting the TAK1-TAB1 chimera (PDB: 5V5N), as TAK1 depends on the binding protein TAB1 for complete activation. Structurally, TAK1 features an essential kinase domain at its N-terminal region and a C-terminal tail (Brown et al., 2005). Within the kinase domain lies a conserved activation loop (Ala179-Lys190), which facilitates conformational rearrangements required for TAK1 autophosphorylation. It is noteworthy that takinib, a well-characterized TAK1 inhibitor, is known to bind precisely to this activation loop (Totzke et al., 2017). The binding site for the TAK1-OGP1-1 complex was also identified within this groove. The computed binding energy for TAK1 and OGP1-1 was −8.58 kcal/mol, reflecting a high binding affinity. Analysis of docking conformations (Fig. 7A & B) revealed that OGP1-1 engages with TAK1 mainly via hydrophobic forces, electrostatic interactions, and hydrogen bonding. Specifically, OGP1-1 binds within a hydrophobic groove composed of seventeen residues, including Ile75, Val76, Thr178, Ala179, Asp181, Ile182, Gln183, His185, Met186, Thr187, Ala193, Met196, Phe201, Glu202, Ala236, Met240, and Trp241. Among these, four hydrophobic residues, Ile75, Val76, Ile182, and Trp241, contribute substantially to the interaction. Further stabilization is provided by three hydrogen bonds between hydroxyl groups of OGP1-1 and TAK1 residues Lys72, Arg155, and His244 (Fig. 7B). In contrast, the reference compound takinib engages a smaller set of residues primarily through hydrophobic contacts with Val42, Tyr106, and Gly110, along with hydrogen bonds to Lys63 and Ala107 (Totzke et al., 2017). The broader interaction interface occupied by OGP1-1, particularly within the activation loop, suggests enhanced binding stabilization compared to takinib. Beyond takinib, another well-characterized TAK1 inhibitor, 5Z-7-oxozeaenol, binds covalently to the ATP-binding pocket and exhibits potent anti-inflammatory activity in preclinical models (Benedik et al., 2025). However, its lack of selectivity has limited clinical translation. OGP1-1, in contrast, is a polypeptide that engages a broader hydrophobic groove including the activation loop, potentially offering higher selectivity. This unique binding mode may reduce off-target effects, making OGP1-1 an attractive scaffold for developing safer TAK1-targeted anti-inflammatory agents. Collectively, these results demonstrate that hydrophobic interactions and hydrogen bonding are key mechanisms underlying the recognition of TAK1 by OGP1-1.

Subsequently, MD simulations were applied to assess the structural stability and interaction dynamics of the TAK1-OGP1-1 complex. MD simulations represent a computational approach for investigating the time-dependent behavior of molecular systems through numerical integration of Newton's equations of motion. This methodology offers valuable information regarding molecular interactions, conformational dynamics, and complex stability under near-physiological conditions (Durrant & McCammon, 2011). In the current study, all-atom MD simulations spanning 100 ns were employed to examine the critical binding behavior of OGP1-1, an anti-inflammatory polypeptide, with the TAK1 protein (Fig. 7C).

Root mean square deviation (RMSD) serves as a critical metric in MD simulations, quantifying the conformational deviation of a protein from its initial structure and reflecting the attainment of equilibrium. As shown in Fig. 7D, the MD trajectories of both the TAK1 alone and the TAK1-OGP1-1 complex systems were analyzed over a 100 ns period. In the complex system, the backbone RMSD of TAK1 exhibited considerable fluctuations during the first 60 ns, suggesting that structural rearrangements occurred to establish a more stable binding interface with OGP1-1. After this period, the RMSD values plateaued, indicating the formation of a stable complex (Zhai et al., 2025).

Root mean square fluctuation (RMSF) analysis of the final 100 ns trajectories revealed increased flexibility in key regions of TAK1 upon OGP1-1 binding, particularly within the activation loop (residues Ala179-Lys190, Fig. 7E). This implies that interactions with OGP1-1 induced local conformational adjustments, enhancing residue mobility, a phenomenon consistent with earlier reports on TAK1-ligand binding (Chuanphongpanich et al., 2023).

The radius of gyration (Rg), which reflects overall structural compactness, was higher in the TAK1-OGP1-1 system than in apo-TAK1 throughout the simulation (Fig. 7F), suggesting a less compact and more expanded conformation upon ligand binding. This observation was supported by the solvent-accessible surface area (SASA) results, which indicated sustained conformational stability in the complex (Fig. 7G).

MM-GBSA calculations performed on the final 100 ns trajectories yielded a total binding free energy (Eₜ) of −44.39 kJ/mol for the complex (Fig. 7H), indicating spontaneous and strong binding (Y. Li, J. Xu, et al., 2024). The energy decomposition revealed that van der Waals interactions (Eᵥ) were the major favorable contributor (−102.63 kJ/mol), followed by electrostatic (Eₑ) and nonpolar solvation (Eₛ) terms. In contrast, the polar solvation energy (Eₚ) opposed complex formation. The significant contribution of E_s_, associated with hydrophobic effects, underscores the role of hydrophobic driving forces in the binding mechanism, aligning with previous studies on protein-peptide systems (Gong et al., 2025).

Residue-wise decomposition analysis further identified key binding contributors, including Lys72, Ile75, Val76, Thr178, Ala179, Ile182, Thr184, His185, Thr187, and Asn188. Among these, Lys72, Arg155, and His244 formed hydrogen bonds critical for complex stability, while Ile75, Val76, and Ile182 primarily provided van der Waals stabilization (Fig. 7B & I). These results offer molecular-level insights into the interaction between OGP1-1 and TAK1.

### 3.10 OGP1-1 exhibited anti-inflammation via TAK1 mediated MAPK and NF-κB pathway

To further elucidate the mechanism by which OGP1-1 suppresses TAK1 activity and exerts its anti-inflammatory action, we focused on key signaling pathways involved in inflammation. The MAPK cascade is known to enhance the production of proinflammatory cytokines such as TNF-α, IL-6, and IL-1β within immune cells. Naturally derived bioactive polypeptides have been reported to attenuate inflammatory responses through inhibition of MAPK signaling (Lou et al., 2025; Xin et al., 2025). Similarly, NF-κB, a transcription factor critical for initiating innate immune responses and regulating inflammatory gene expression, requires nuclear translocation to exert its effects (T. Liu et al., 2017). Persistent activation of NF-κB is linked to chronic inflammatory diseases, underscoring the importance of its regulation. Moreover, TAK1 has been established as a key upstream kinase governing both MAPK and NF-κB pathways. Considering above results in this study, we proposed that OGP1-1 alleviates inflammation in LPS-stimulated macrophages by modulating the TAK1-dependent MAPK-NF-κB axis.

**Fig. 8:**
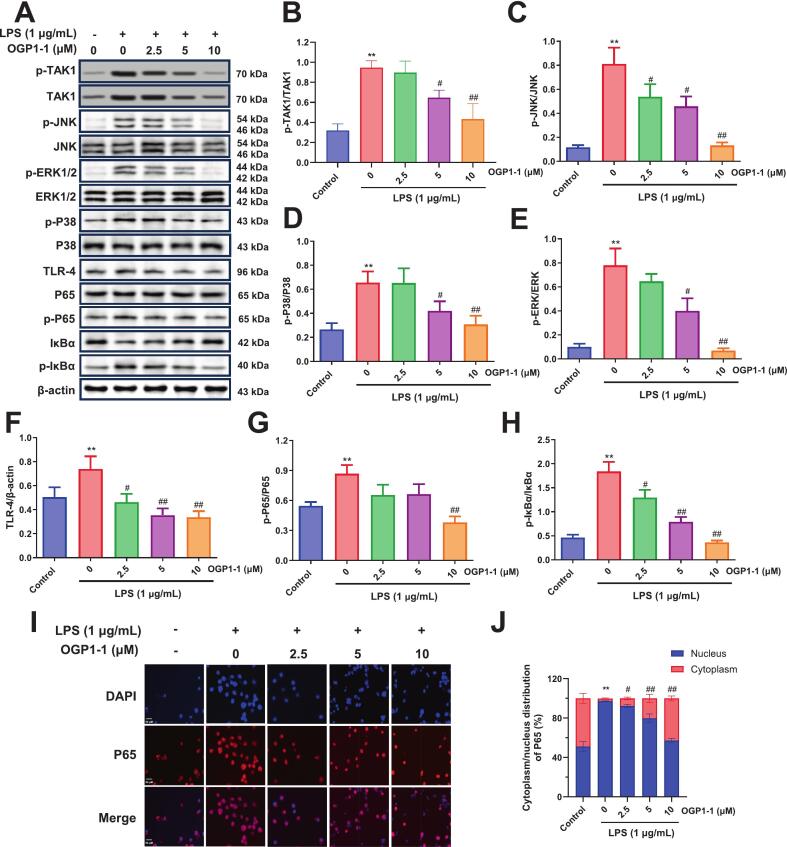
OGP1-1 treatment modulated MAPK and NF-κB pathways in LPS-stimulated RAW264.7 macrophages. (A) Western blotting and immunostaining were conducted for analysis of TAK1 and TLR4 expression, MAPK phosphorylation and translocation of NF-κB into the nucleus. (B) relative levels of p-TAK1/TAK1 protein, (C) relative levels of p-JNK/JNK protein, (D) relative levels of p-P38/P38 protein, (E) relative levels of p-ERK/ERK protein, (F) relative levels of TLR4 protein, (G) relative levels of p-P65/P65 protein, (H) relative levels of p-IκBα/IκBα protein and (I) immunostaining of NF-κB. (J) Quantitative analysis of NF-κB p65 nuclear translocation in (I). Data in (B), (C), (D), (F), (G), (H), and (J) are presented as the means ± SD (**P < 0.01 vs. the control group; ##*P* < 0.01 vs. LPS group, *n* = 3 each group).

Western blot analysis revealed that LPS challenge markedly enhanced phosphorylation of TAK1 and the MAPKs JNK, ERK, and p38 in RAW264.7 cells (Fig. 8A). Pre-incubation with OGP1-1 concentration-dependently suppressed the phosphorylation of these kinases, confirming its role in mitigating MAPK activation (Fig. 8B–D). Subsequent evaluation of NF-κB p65 subcellular localization via western blot and immunofluorescence staining showed that LPS promoted robust nuclear translocation of p65, which was markedly attenuated by OGP1-1 treatment (Fig. 8E–H). Concomitantly, the decline in cytosolic NF-κB p65 induced by LPS was reversed upon OGP1-1 exposure, consistent with inhibition of NF-κB signaling. Immunofluorescence quantification further supported these observations, demonstrating a significant reduction in nuclear p65 intensity following OGP1-1 administration (Fig. 8I, J). Collectively, these data substantiate that OGP1-1 mediates its anti-inflammatory effects primarily through interference with the TAK1-MAPK-NF-κB pathway.

The cupin protein superfamily exhibits remarkable functional diversity among recognized protein groups. These proteins are distinguished by their consistent β-barrel architecture and two distinct short signature sequence motifs: GX_5_HXHX_3, 4_E (or D) X_6_G, and GX_5_PXGX_2_HX_3_N. These motifs contain residues that bind to metal ions. A sequence of 15–50 amino acids exists between the two motifs (Hu et al., 2023). The biological functions of cupin proteins are elucidated by the binding of various metal ions, including Fe^2+^, Mn^2+^, Ni^2+^, Cu^2+^, Zn^2+^, and Cd^2+^, to the cupin active site (Agarwal et al., 2009). They exhibit diverse enzymatic activities, such as dioxygenases, isomerases, and oxalate oxidases, alongside non-enzymatic activities, including auxin binding, sucrose binding, seed storage, and transcriptional factors, in archaea, eubacteria, and eukaryota. While the initial focus was on the physiological activities of cupin proteins, current research indicates that this superfamily may potentially be implicated in human illnesses (T. Wang et al., 2023). Pirin, a cupin domain containing protein, is extensively conserved among mammals, plants, fungi, and prokaryotes. The crystal structure of pirin has similarities to cupin proteins, with two antiparallel germin-like β-barrel domains, two distinct cupin family motifs, a single Fe^2+^ in the N-terminal domain, and a C-terminal region featuring one α-helix (Barman & Hamelberg, 2016). Pirin has recently been recognized as a transcriptional co-regulator. Pirin interacts with the transcription factors NF-I and BCL-3, creating complexes with NF-κB p50. Pirin functions as an iron-dependent regulator of oxidative stress. The structural transition between Fe^2+^ (inactive) and Fe^3+^ (active) oxidation states under oxidative circumstances modulate its conformation and affinity for NF-κB p65 (F. Liu et al., 2013). NF-κB is a pervasive transcriptional regulator deemed essential in immune response and inflammation, however, the anti-inflammatory effects of pirin remain unexplored. Despite the strong correlation between cupin domain-containing polypeptides and inflammation, there has been little study on the anti-inflammatory action of cupin domain-derived polypeptides on the human innate immune system. To our knowledge, this is the first report of a cupin domain-containing polypeptide demonstrating anti-inflammatory activity.

## 4 Conclusions

In summary, we have discovered a novel polypeptide, OGP1-1, from oyster, *Ostrea gigas*. According to the identification of amino acid sequence, OGP1-1 showed moderate identity to known cupin domain-containing polypeptides in NCBI nr database, and thereby established its classification as a novel cupin-like polypeptide in *O. gigas*. Physicochemical properties and structure analysis of OGP1-1 were revealed by electrophoresis, UV–vis absorption, FT-IR and CD spectroscopy. OGP1-1 demonstrated significant efficacy in alleviating inflammatory responses both in vitro and in vivo, by effectively downregulating the production of key inflammatory mediators, including NO, TNF-α, and IL-1β. Notably, TAK1 was identified as the direct molecular target of OGP1-1, confirmed through advanced chemical proteomics and biophysical binding assays. The interaction was further elucidated at the atomic level via molecular docking and dynamics simulations, which confirmed a stable binding mode primarily driven by hydrophobic interactions and hydrogen bonding within the kinase domain. The subsequent mechanistic investigation established that OGP1-1 exerts its potent anti-inflammatory effects by specifically inhibiting the TAK1-dependent activation of the downstream MAPK (JNK, p38, ERK) and NF-κB pathways, thereby preventing the nuclear translocation of NF-κB p65. Collectively, these findings indicate that OGP1-1 is a polypeptide with significant immunological activity, and highlight the vast potential of underutilized seafood as a source of high-value bioactive polypeptides for application in functional foods aimed at managing chronic inflammation.

## CRediT authorship contribution statement

**Chunlei Li:** Writing – original draft, Supervision, Investigation, Conceptualization. **Yanxiao Xiang:** Investigation, Formal analysis, Data curation. **Tao Jiang:** Investigation, Formal analysis. **Jiyuan Zhang:** Investigation, Formal analysis. **Xuekui Xia:** Writing – review & editing, Supervision, Resources. **Anchang Liu:** Writing – review & editing, Supervision, Resources, Project administration.

## Declaration of competing interest

The authors declare that they have no known competing financial interests or personal relationships that could have appeared to influence the work reported in this paper.

## Data Availability

Data will be made available on request.
