# Supplementary material for: Screening and characterization of a novel cupin-like polypeptide from Oyster (Ostrea gigas) and its anti-inflammatory effect via TAK1 mediated NF-κB pathway
Source: Food Chem X. 2026 Apr 21;36:103884. doi: 10.1016/j.fochx.2026.103884 (PMC13129452; doi:10.1016/j.fochx.2026.103884)
Supplement: Supplementary material [file mmc1.docx]

**Supporting information**

**Screening and characterization of a novel cupin-like polypeptide from Oyster (*Ostrea gigas*) and its anti-inflammatory effect via TAK1 mediated NF-κB pathway**

Chunlei Li^a,b,1^, Yanxiao Xiang^a,1^, Tao Jiang^b^, Jiyuan Zhang^b^, Xuekui Xia^b*^ and Anchang Liu^a,c*^

*^a^ Department of Pharmacy, Qilu Hospital, Cheeloo College of Medicine, Shandong University, Jinan 250012, China.*

*^b^ Shandong Provincial Key Laboratory for Bio-Manufacturing, Biology Institute, Qilu University of Technology (Shandong Academy of Sciences), Jinan 250103, China.*

*^c^ Department of Clinical Pharmacy, School of Pharmaceutical Sciences, Shandong University, Jinan, China.*

*^1^* These authors contribute equally.

***** Corresponding authors, E-mail addresses: xiaxk@sdas.org (X. Xia), anchang_liu@126.com (A. Liu)

**Table S1.** qPCR primers required for this study.

| **Species** | **Gene Name (Accession No.)** | | **Sequence（5’-3’）** | **Amplicon length (bp)** |
| --- | --- | --- | --- | --- |
| zebrafish | *β-actin*  (NM_131031.2) | Forward | AGAGCTATGAGCTGCCTGACG | 106 |
|  |  | Reverse | CCGCAAGATTCCATACCCA |  |
| zebrafish | *NOS2b*  (NM_001113501.1) | Forward | TGCCGCCTATGTCTGGATCTCTG | 117 |
|  |  | Reverse | TGTCCTTCCACTTGTGCGTCAAC |  |
| zebrafish | *TNF-α*  (NM_212859.2) | Forward | GCAATCCGCTCAATCTGCAC | 127 |
|  |  | Reverse | GAAGTGCTGTGGTCGTGTCT |  |
| zebrafish | *IL-1β*  (NM_212844.2) | Forward | ATGGAGAGCTGCAGACCAAC | 106 |
|  |  | Reverse | GCATCAGTGTGTGTGCTGTG |  |
| zebrafish | *COX-2*  (NM_001025504.2) | Forward | ACCTTTGGTGGGAAAGTGGG | 108 |
|  |  | Reverse | AGGCACATAAAAGGACGCCA |  |
| mouse | *iNOS*  (NM_010927.4) | Forward | TTCCAGAATCCCTGGACAAG | 177 |
|  |  | Reverse | TGGTCAAACTCTTGGGGTTC |  |
| mouse | *IL-6*  (NM_031168.2) | Forward | GGAGTCACAGAAGGAGTGGC | 109 |
|  |  | Reverse | AACGCACTAGGTTTGCCGAG |  |
| mouse | *TNF-α*  (NM_013693.3) | Forward | GGACAGTGACCTGGACTGTG | 127 |
|  |  | Reverse | GAGGCAACCTGACCACTCTC |  |
| mouse | *IL-1β*  (NM_008361.4) | Forward | AAGGGGACATTAGGCAGCAC | 91 |
|  |  | Reverse | ATGAAAGACCTCAGTGCGGG |  |
| mouse | *β-actin*  (NM_007393.5) | Forward | TAAGAGGAGGATGGTCGCGT | 153 |
|  |  | Reverse | CTCAGACCTGGGCCATTCAG |  |

**Table S2.** Amino acid sequence analysis of OGP1-1 protein by tandem mass spectrometry.

| **Protein FDR Confidence** | **Accession** | **-10lgP** | **Coverage (%)** | **Area OGP1-1** | **#Unique** | **#PSMs** | **# Protein Groups** | **#AAs** | **Avg. Mass** | **calc. pI** | **Score Sequest HT** |
| --- | --- | --- | --- | --- | --- | --- | --- | --- | --- | --- | --- |
| High | XM_034448153.1 | 418.59 | 98 | 5.31E+07 | 54 | 690 | 1 | 101 | 11701.44 | 9.07 | 1605.336 |
| **Peptide** | **-10lgP** | **Mass** | **Length** | **ppm** | **m/z** | **RT** | **Area OGP1-1** | **Fraction** | **Scan** | **#PSMs** | **PTM** |
| HFPDHTHGVSKKDAILRGK | 143.63 | 2143.4471 | 19 | 1.4 | 715.4806 | 6.82 | 2.78E+07 | 1 | 37042 | 313 |  |
| WNEEKDGKLSESSLEQK | 105.19 | 2007.1458 | 16 | 3.7 | 670.0482 | 7.47 | 3.86E+06 | 1 | 37680 | 13 |  |
| VEKWNEEKDGKLSESSLEQK | 105.1 | 2363.5674 | 20 | 3.8 | 788.8559 | 7.19 | 1.72E+06 | 1 | 37404 | 9 |  |
| WN(+.98)EEKDGKLSESSLEQ(+.98)K | 99.57 | 2009.1007 | 17 | 2.2 | 670.7089 | 6.29 | 2.51E+06 | 1 | 36502 | 13 | Deamidation (NQ) |
| HFPDHTHGVSKKDAILRGQMK | 94.81 | 2402.7604 | 21 | -0.4 | 601.6971 | 7.54 | 2.38E+05 | 1 | 37747 | 4 |  |
| PDHTHGVSKKDAILRGQMK | 92.99 | 2118.4409 | 19 | -0.2 | 707.1441 | 6.45 | 9.01E+04 | 1 | 36654 | 4 |  |
| HFPDHTHGVSKK | 92.44 | 1389.5408 | 12 | 1.6 | 464.1883 | 6.27 | 8.37E+05 | 1 | 36485 | 5 |  |
| HFPDHTHGVSKKDAILRGQM(+15.99)K | 83.36 | 2418.7656 | 21 | 0.6 | 605.6965 | 5.65 | 6.37E+05 | 1 | 35871 | 3 | Oxidation (M) |
| KHFPDHTHGVSKKDAILRGQMK | 83.31 | 2530.932 | 22 | -2.6 | 507.1788 | 5.77 | 9.91E+04 | 1 | 35986 | 3 |  |
| VEKWN(+.98)EEKDGKLSESSLEQ(+.98)K | 82.03 | 2363.5622 | 20 | 2.8 | 788.8594 | 6.1 | 1.16E+06 | 1 | 36312 | 11 | Oxidation (M) |
| HFPDHTHGVSK | 78.66 | 1261.3615 | 11 | 6.8 | 421.4551 | 5.16 | 3.64E+04 | 1 | 35390 | 4 |  |
| VEKWNEEKDGKL | 77.99 | 1474.6318 | 12 | 2.6 | 492.5452 | 7.41 | 1.18E+05 | 1 | 37616 | 2 |  |
| W(+42.01)NEEKDGKLSESSLEQK | 75.46 | 2049.1413 | 17 | 3.2 | 684.0431 | 7.25 | 2.34E+04 | 1 | 37465 | 1 | Acetylation (Protein N-term) |
| FCM(+15.99)YGQTVILGPGDM(+15.99)IQVPQDRVH | 74.96 | 2737.1631 | 24 | 1 | 685.2958 | 14.73 | 1.12E+06 | 1 | 44743 | 13 | Oxidation (M) |
| YDFPPGKHFPDHTHGVSKK | 74.94 | 2194.4478 | 19 | 5 | 732.4845 | 6.56 | 2.39E+04 | 1 | 36775 | 2 |  |
| FCMYGQTVILGPGDM(+15.99)IQVPQDRVH | 73.74 | 2721.168 | 24 | -1 | 681.2957 | 12.04 | 5.96E+05 | 1 | 42143 | 9 | Oxidation (M) |
| TVVGKDFLVFYDATR | 73.21 | 1730.9818 | 15 | 3 | 577.9908 | 11.38 | 1.91E+05 | 1 | 41505 | 2 |  |
| FCMYGQTVILGPGDMIQVPQDRVH | 71.93 | 2705.1682 | 24 | -1.7 | 902.7219 | 16.62 | 6.31E+04 | 1 | 46543 | 2 |  |
| DAILRGQMK | 71.82 | 1031.247 | 9 | 6.8 | 344.7426 | 7.02 | 2.26E+05 | 1 | 37230 | 5 |  |
| FCMYGQTVILGPGDMIQVPQDRVHNA | 67.92 | 2890.3456 | 26 | -0.3 | 723.5888 | 14.62 | 1.67E+05 | 1 | 44636 | 4 |  |
| YDFPPGKHFPDHTHGVSKKDAILRGQMK | 69.86 | 3207.6633 | 28 | 3.3 | 642.5379 | 8.82 | 3.47E+05 | 1 | 39006 | 6 |  |
| DFLVFYDATR | 69.35 | 1246.3998 | 10 | 4.9 | 416.4662 | 10.52 | 1.00E+05 | 1 | 40672 | 4 |  |
| FCM(+15.99)YGQTVILGPGDM(+15.99)IQVPQDRVHNA | 67.92 | 2922.3342 | 26 | -0.3 | 975.1188 | 14.62 | 1.67E+05 | 1 | 44636 | 4 | Oxidation (M) |
| YDFPPGKHFPDHTH | 67.15 | 1694.8368 | 14 | 2.4 | 565.9424 | 5.9 | 2.38E+04 | 1 | 36112 | 2 |  |
| FC(+57.02)MYGQTVILGPGDMIQVPQDRVHNA | 67 | 2947.362 | 26 | -0.4 | 983.4571 | 16.58 | 2.29E+04 | 1 | 46508 | 2 | Carbamidomethylation |
| FCMYGQTVILGPGDM(+15.99) | 65.97 | 1647.9449 | 15 | 0.7 | 550.3153 | 13.76 | 1.41E+04 | 1 | 43803 | 1 | Oxidation (M) |
| FCM(+15.99)YGQTVILGPGDMIQVPQDRVH | 65.64 | 2705.1619 | 24 | 3.7 | 902.7288 | 12.53 | 2.74E+05 | 1 | 42623 | 6 | Oxidation (M) |
| VVGKDFLVFYDATR | 64.18 | 1629.889 | 14 | 5.1 | 544.2989 | 14.09 | 3.05E+04 | 1 | 44122 | 2 |  |
| FCMYGQ(+.98)TVILGPGDM(+15.99)IQ(+.98)VPQ(+.98)DRVHN(+.98)A | 63.77 | 2909.3027 | 26 | 6.4 | 728.3244 | 16.16 | 3.35E+04 | 1 | 46114 | 3 | Oxidation (M); Deamidation (NQ) |
| FC(+57.02)M(+15.99)YGQTVILGPGDM(+15.99)IQVPQDRVHNA | 63.58 | 2979.3402 | 26 | 2 | 745.8327 | 12.2 | 3.19E+05 | 1 | 42295 | 4 | Oxidation (M); Carbamidomethylation |
| VHNATVVGKDFLVFYDATR | 63.15 | 2152.4469 | 19 | 0.2 | 718.4831 | 12.86 | 3.06E+04 | 1 | 42935 | 2 |  |
| DHTHGVSKKDAILRGQMK | 61.27 | 2021.3389 | 18 | 5.7 | 674.7772 | 5.83 | 2.77E+04 | 1 | 36042 | 1 |  |
| VHN(+.98)ATVVGKDFLVFYDATR | 60.72 | 2153.4218 | 19 | 2.2 | 718.8063 | 10.05 | 7.06E+04 | 1 | 40208 | 1 | Deamidation (NQ) |
| QGYKFIRYDFPPGK | 59.81 | 1715.9774 | 14 | 2.8 | 572.9914 | 11.67 | 9.54E+04 | 1 | 41772 | 4 |  |
| QGYKFIR | 59.51 | 911.0712 | 7 | 8.5 | 304.6969 | 7.78 | 9.00E+05 | 1 | 37985 | 47 |  |
| FCM(+15.99)YGQTVILGPGDM(+15.99)IQVPQDR | 59.01 | 2500.8956 | 22 | 2.7 | 834.6317 | 14.32 | 1.67E+05 | 1 | 44342 | 2 | Oxidation (M) |
| FCMYGQTVILGPGDMIQVPQDR | 58.37 | 2468.8907 | 22 | -0.1 | 823.9607 | 19.05 | 6.27E+04 | 1 | 48829 | 4 |  |
| FCM(+15.99)YGQTVILGPGDMIQVPQDR | 57.86 | 2484.8704 | 22 | 4.9 | 829.2976 | 14.27 | 7.63E+05 | 1 | 44295 | 13 | Oxidation (M) |
| FC(+57.02)M(+15.99)YGQTVILGPGDM(+15.99)IQVPQDR | 57.21 | 2557.9056 | 22 | 4.9 | 853.6357 | 14.94 | 5.78E+05 | 1 | 44940 | 2 | Oxidation (M); Carbamidomethylation |
| THGVSKKDAILRGQMK | 54.03 | 1769.1031 | 16 | 1.8 | 590.7087 | 5.93 | 6.81E+03 | 1 | 36146 | 1 |  |
| HFPDHTHGVSKKDAILRGQ(+.98)MK | 52.78 | 2403.741 | 21 | 9.9 | 802.2446 | 6.29 | 3.52E+06 | 1 | 36504 | 4 | Deamidation (NQ) |
| GQMKFCMYGQTVILGPGDMIQVPQDR | 51.97 | 2913.4464 | 26 | -2.9 | 729.3633 | 15.14 | 3.83E+03 | 1 | 45140 | 1 |  |
| GQM(+15.99)KFCMYGQTVILGPGDMIQVPQDRVH | 50.89 | 3165.7178 | 28 | -1.4 | 634.1415 | 14.14 | 1.76E+05 | 1 | 44165 | 5 | Oxidation (M); Carbamidomethylation |
| GQM(+15.99)KFCMYGQTVILGPGDMIQVPQDR | 50.71 | 2929.437 | 26 | 4.3 | 600.8767 | 15.21 | 0 | 1 | 45211 | 3 | Oxidation (M) |
| GQM(+15.99)KFC(+57.02)MYGQTVILGPGDMIQVPQDR | 50.27 | 2986.452 | 26 | 1 | 747.6176 | 15.89 | 3.41E+04 | 1 | 45858 | 2 | Oxidation (M); Carbamidomethylation |
| YDFPPGK | 48.79 | 822.9268 | 7 | 2 | 275.3015 | 5.61 | 2.21E+05 | 1 | 35824 | 65 |  |
| YDFPPGKHF | 46.88 | 1107.2329 | 9 | 1 | 370.0793 | 11.14 | 4.92E+05 | 1 | 41268 | 10 |  |
| DGKLSESSLEQKLRR | 46.04 | 1745.9542 | 15 | 5.2 | 582.9805 | 5.86 | 4.42E+03 | 1 | 36074 | 1 |  |
| FCM(+15.99)YGQTVILGPG | 45.99 | 1385.6674 | 13 | 5.3 | 462.8846 | 10.04 | 0 | 1 | 40196 | 1 | Oxidation (M) |
| HFPDHTHGVSKKDAILR | 45.93 | 1958.2185 | 17 | 4.5 | 653.7315 | 8.37 | 1.97E+05 | 1 | 38564 | 2 |  |
| FCMYGQTVILGPGDM(+15.99)IQVPQDR | 44.67 | 2484.8704 | 22 | -1.8 | 622.2111 | 17.02 | 3.59E+05 | 1 | 46927 | 9 | Oxidation (M) |
| DAILRGQM(+15.99)K | 43.85 | 1047.2412 | 9 | 3.2 | 350.0814 | 10.11 | 9.39E+03 | 1 | 40264 | 1 | Oxidation (M) |
| HTHGVSKKDAILRGQMK | 43.49 | 1906.2419 | 17 | 17.7 | 636.4101 | 5.53 | 0 | 1 | 35750 | 1 |  |
| GQM(+15.99)KFCM(+15.99)YGQTVILGPGDMIQVPQDR | 51.97 | 2945.4113 | 26 | -2.9 | 737.3533 | 15.14 | 3.83E+03 | 1 | 45140 | 1 | Oxidation (M) |
| NATVVGKDFLVFYDATR | 43 | 1916.1696 | 17 | -1.2 | 639.7297 | 15.05 | 7.33E+03 | 1 | 45052 | 1 |  |
| GQM(+15.99)KFCM(+15.99)YGQTVILGPGDM(+15.99)IQVPQDR | 40.71 | 2961.404 | 26 | -1.2 | 988.1379 | 15.18 | 1.23E+05 | 1 | 45180 | 3 | Oxidation (M) |
| DAILRGQMKFC | 40.03 | 1281.5594 | 11 | 4.6 | 428.1843 | 13.44 | 3.80E+03 | 1 | 43493 | 1 |  |
| FCMYGQTVILGPGDM | 39.13 | 1631.949 | 15 | 12.9 | 544.9875 | 16.73 | 3.61E+03 | 1 | 46648 | 1 |  |
| DGKLSESSLEQK | 37.29 | 1320.4214 | 12 | 5.5 | 441.1479 | 5.02 | 7.88E+02 | 1 | 35256 | 1 |  |
| LSESSLEQKLR | 37.13 | 1289.4572 | 11 | -7.5 | 430.8116 | 7.07 | 1.14E+04 | 1 | 37281 | 1 |  |
| GQM(+15.99)KFC(+57.02)M(+15.99)YGQTVILGPGDMIQVPQDR | 36.53 | 3002.434 | 26 | -4.7 | 601.4075 | 13.24 | 3.06E+04 | 1 | 43307 | 1 | Oxidation (M); Carbamidomethylation |
| FCMYGQ | 36.3 | 747.8834 | 6 | 1.7 | 250.2946 | 10.21 | 3.59E+03 | 1 | 40359 | 1 |  |
| PDHTHGVSKKDAILRGQM(+15.99)K | 32.59 | 2124.4242 | 19 | -18.7 | 709.1481 | 6.37 | 8.05E+04 | 1 | 36582 | 1 | Oxidation (M) |
| WNEEKDGK | 31.47 | 1005.0542 | 8 | 4.7 | 336.0171 | 9.15 | 0 | 1 | 39326 | 1 |  |
| VEKWNEEK | 29.37 | 1061.1638 | 8 | 1.1 | 354.7247 | 9.62 | 9.12E+03 | 1 | 39783 | 2 |  |
| PDHTHGVSKKDAILR | 29.03 | 1673.8983 | 15 | -12.7 | 558.9675 | 6.8 | 6.01E+05 | 1 | 37017 | 5 |  |
| DFPPGKHFPDHTHGVSKKDAILRGQMK | 29.02 | 3044.4872 | 27 | 6.6 | 609.8966 | 9 | 1.14E+05 | 1 | 39182 | 1 |  |
| DGELNSENM(+15.99)EK | 28.09 | 1280.519 | 11 | 10.6 | 427.8336 | 5.07 | 9.76E+03 | 1 | 35310 | 1 | Oxidation (M) |
| Q(-17.03)GYKFIR | 27.88 | 894.0426 | 7 | 3.6 | 299.0151 | 11.81 | 1.20E+04 | 1 | 41906 | 2 | Pyro-glu from Q |
| Q(-17.03)GYKFIRYDF | 21.72 | 1336.5105 | 10 | -3.5 | 446.5051 | 12.81 | 9.15E+03 | 1 | 42886 | 1 | Pyro-glu from Q |

**Table S3.** Minimum Information for Publication of Quantitative Real-Time PCR Experiments **(**MIQE) checklist for qPCR analysis

| **Item** | **Importance** | **Provided** | **Description/Justification** |
| --- | --- | --- | --- |
| **1. EXPERIMENTAL DESIGN** | | | |
| **Definition of experimental and control groups** | E | Yes | RAW264.7 macrophages: Control group (untreated), LPS model group (1 μg/mL LPS), treatment groups (OGP1-1 at 2.5, 5, or 10 μM with LPS). All non-control cultures received 30 minutes of pre-exposure to LPS before the introduction of OGP1-1, followed by a 24-hour incubation period. See Section 2.13.  Transgenic zebrafish: 50 juvenile zebrafish (n=50 per group). The experimental groups included: (1) untreated controls (fresh culture medium), (2) a positive control (20 μM ibuprofen), and (3) test groups (2.5, 5, or 10 μM samples). Following a 6-hour pretreatment, all groups except controls were exposed to 20 μM CuSO4 in darkness for 1 hour. See Section 2.24. |
| **Number within each group** | E | Yes | Three independent biological replicates (n=3). See Section 2.13 & 2.24. |
| **Assay carried out by core lab or investigator's lab** | D | Yes | Investigator's lab. |
| **2. SAMPLE** | | | |
| **Description** | E | Yes | RAW264.7 macrophages and zebrafish larvae. See Section 2.13 (cells) and Section 2.24 (zebrafish) |
| **Volume/mass of sample processed** | D | Yes | 1 × 10⁶ cells/mL for macrophages; 50 juvenile zebrafish per group. See Section 2.13 & 2.24. |
| **Processing procedure** | E | Yes | Trizol reagent extraction. See Section 2.13 & 2.24. |
| **Sample storage conditions and duration** | E | Yes | Total RNA stored at −80°C for 48 h. See Section 2.24 |
| **3. NUCLEIC ACID EXTRACTION** | | | |
| **Procedure and/or instrumentation** | E | Yes | Trizol reagent (Aladdin, Shanghai, China) and FastPure cell/tissue total RNA extraction kit (Vazyme). See Section 2.1. |
| **DNase treatment** | E | Yes | Included in HiScript II Q RT SuperMix with gDNA wiper (Vazyme). See Section 2.1. |
| **Contamination assessment (DNA)** | E | Yes | gDNA wiper included in the reverse transcription kit (HiScript II Q RT SuperMix, Vazyme) to remove residual genomic DNA; no-RT controls were included and showed no amplification (Cq > 35). |
| **Nucleic acid quantification** | E | Yes | Spectrophotometric quantification. See Section 2.13. |
| **Purity (A260/A280)** | D | Yes | A260/A280 ratio measured by Nanodrop spectrophotometer; values ranged from 1.8 to 2.0 for all samples, indicating high RNA purity. |
| **RNA integrity method/instrument** | E | Yes | Gel electrophoresis (1.2% agarose gel, 180 V for 30 min); distinct 28S and 18S rRNA bands observed with approximate 2:1 intensity ratio, indicating intact RNA. |
| **4. REVERSE TRANSCRIPTION** | | | |
| **Complete reaction conditions** | E | Yes | One-Step PrimeScript RT-PCR Kit (Takara, Japan). See Section 2.13. |
| **Amount of RNA and reaction volume** | E | Yes | 50 μL total reaction volume. See Section 2.13. |
| **Priming method** | E | Yes | Random primers and oligo(dT) primers mixture (provided in One-Step PrimeScript RT-PCR Kit, Takara). |
| **Reverse transcriptase and concentration** | E | Yes | PrimeScript Reverse Transcriptase (provided in One-Step PrimeScript RT-PCR Kit, Takara)；concentration as per manufacturer's protocol. See Section 2.13. |
| **Manufacturer of reagents** | D | Yes | Takara Corporation (Shiga, Japan). See Section 2.1 and 2.13. |
| **cDNA storage conditions** | D | Not applicable | cDNA was used immediately for qPCR without storage in the one-step RT-qPCR protocol. |
| **5. qPCR TARGET INFORMATION** | | | |
| **Sequence accession number** | E | Yes | See Table S1. |
| **Amplicon length** | E | Yes | Provided in Table S1. |
| **In silico specificity screen (BLAST)** | E | Yes | In silico specificity was confirmed using NCBI Primer-BLAST with the reference sequences corresponding to the accession numbers listed in Table S1; all primer pairs were verified to amplify only the intended targets. |
| **6. qPCR OLIGONUCLEOTIDES** | | | |
| **Primer sequences** | E | Yes | Provided in Table S1. |
| **Probe sequences** | D | Not applicable | SYBR Green I used; no probe was applied in this experiment. |
| **Manufacturer of oligonucleotides** | D | Yes | All primers were synthesized by Sangon Biotech, Shanghai, China. |
| **7. qPCR PROTOCOL** | | | |
| **Complete reaction conditions** | E | Yes | Total reaction volume: 50 μL, containing 25 μL of 2× AceQ qPCR SYBR Green Master Mix (Vazyme), 0.2 μM each of forward and reverse primers, 2 μL of cDNA template (diluted 1:5 from RT reaction), and RNase-free water to final volume. Cycling conditions: 95 °C for 30 s; 40 cycles of 90 °C for 5 s, 60 °C for 34 s. |
| **Reaction volume and amount of cDNA** | E | Yes | 50 μL; one-step RT-qPCR with 1 μg total RNA input per reaction (Takara One-Step PrimeScript RT-PCR Kit). |
| **Primer concentration** | E | Yes | 0.2 μM each of forward and reverse primers. |
| **Polymerase identity and concentration** | E | Yes | AceTaq DNA Polymerase (hot-start), proprietary concentration as provided in 2× AceQ qPCR SYBR Green Master Mix (Vazyme); final concentration in reaction is 1×. |
| **Buffer/kit identity and manufacturer** | E | Yes | AceQ qPCR SYBR Green master mix (Vazyme Corporation, Nanjing, China). See Section 2.1 |
| **Additives (SYBR Green I)** | E | Yes | SYBR Green I (included in AceQ qPCR SYBR Green Master Mix, Vazyme); no other additives. |
| **Complete thermocycling parameters** | E | Yes | 95°C for 30 s; 40 cycles of 90°C for 5 s, 60°C for 34 s. See Section 2.13. |
| **Manufacturer of qPCR instrument** | E | Yes | Bio-rad CFX 96 Real-Time PCR Detection System (Bia-Rad, Hercules, USA). |
| **8. qPCR VALIDATION** | | | |
| **Specificity (melt curve)** | E | Yes | Melt curve analysis performed; single peak observed for all targets, indicating specific amplification. |
| **For SYBR Green I, Cq of NTC** | E | Yes | NTC Cq > 35 for all primer sets. |
| **Standard curves with slope and y-intercept** | E | Yes | Standard curves were generated for each primer pair using serial dilutions of pooled cDNA. Slopes ranged from –3.32 to –3.45; y-intercept values ranged from 32 to 38. |
| **PCR efficiency calculated from slope** | E | Yes | PCR efficiency ranges from 95 to 105%. The difference in amplification efficiency between target and reference genes was < 5%, validating the use of the 2^⁻ΔΔCt^ method. |
| **R² of standard curve** | E | Yes | R² > 0.99. |
| **9. DATA ANALYSIS** | | | |
| **qPCR analysis program (source, version)** | E | Yes | CFX Maestro v4.1 (Bio-Rad) for data acquisition; GraphPad Prism 9.5 for ΔΔCt calculation and statistics. |
| **Cq method determination** | E | Yes | 2^⁻ΔΔCt^ method. See Section 2.13. |
| **Outlier identification and disposition** | E | Yes | Technical replicates with Cq SD > 0.25 were excluded; all biological replicates (n = 3) were included in analysis. |
| **Results of NTCs** | E | Yes | NTC Cq > 35 for all primer pairs; no significant primer-dimer or contamination observed. |
| **Justification of number and choice of reference genes** | E | Yes | β‑actin was used as the sole reference gene. Its expression stability was validated in preliminary experiments (Cq variation < 0.5 cycles across treatment groups), and previous literature confirms its stability under LPS stimulation in RAW264.7 macrophages and zebrafish models. |
| **Description of normalization method** | E | Yes | Relative expression was calculated using the 2⁻ΔΔCt method, and β‑actin was used as the reference gene. See Section 2.13. |
| **Number and stage of technical replicates** | E | Yes | qPCR reactions were performed in triplicate (3 technical replicates per sample). No technical replicates were performed at the reverse transcription stage. |
| **Repeatability (intra-assay variation)** | E | Yes | Intra-assay variation was assessed by calculating the standard deviation (SD) of Cq values. |
| **Statistical methods for result significance** | E | Yes | One-way ANOVA with *P* < 0.05 considered significant; t-test for two-group comparisons. See Section 2.25. |
| **Software (source, version)** | E | Yes | GraphPad Prism 9.5. See Section 2.25. |


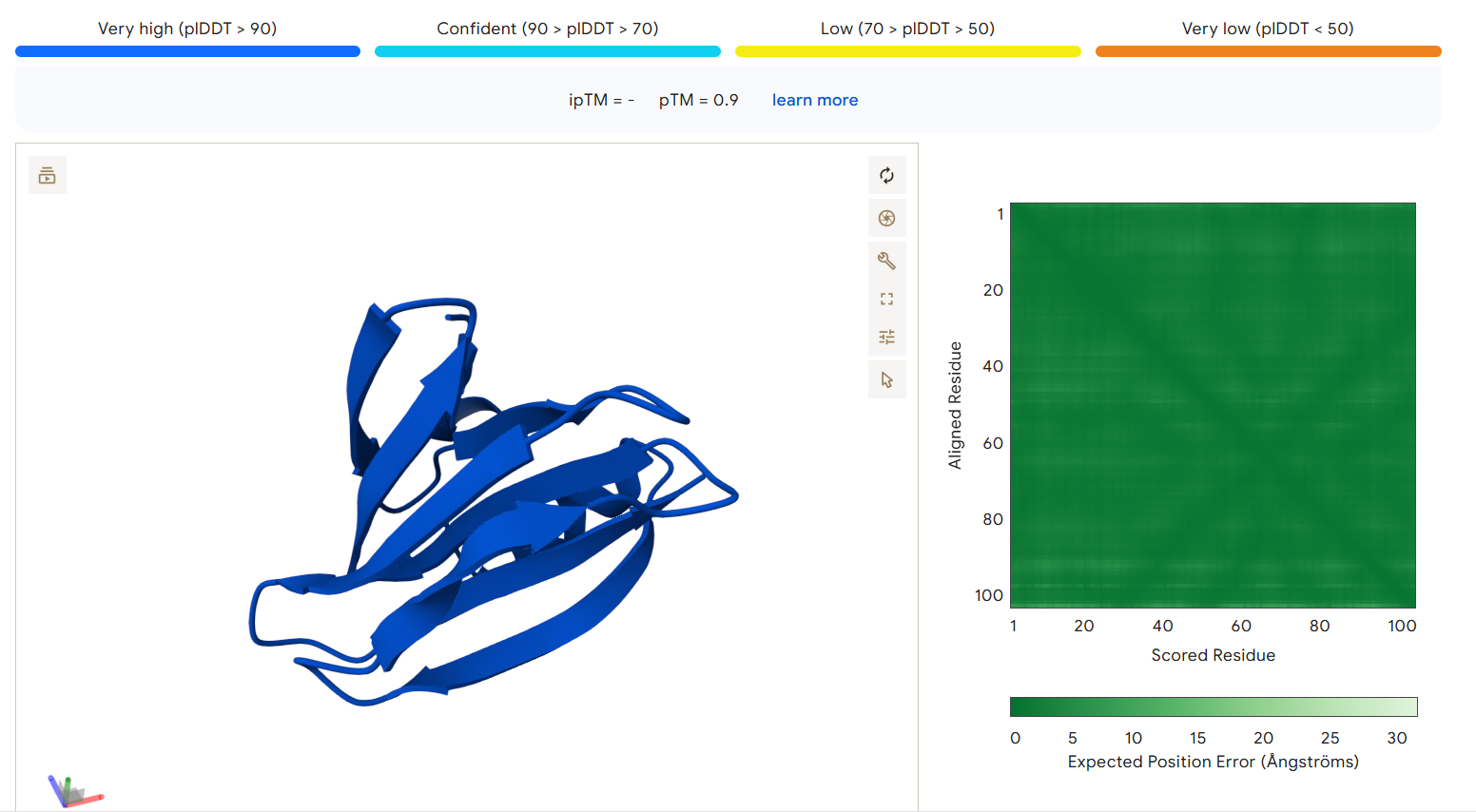

**Fig. S1**. The homology modeling parameters of OGP1-1 generated by AlphaFold online server.

**Fig. S2**. The inhibitory capacity of OGP1-1 against NO production following (A) heat and (B) pH treatments. Values are the means ± SD (n = 3). The mean values denoted by the same letters are not significantly different (*P* > 0.05) according to Student's t-test using GraphPad Prism 9.5.
